# The Physiological Responses of Tea to pH and Cd Conditions and the Effect of the *CsHMA2* on Cd Transport

**DOI:** 10.3390/plants14040570

**Published:** 2025-02-13

**Authors:** Bin Yang, Yao Xiao, Lei Li, Min Shen, Xiaogang Lei, Xujun Zhu, Wanping Fang

**Affiliations:** 1 College of Rural Revitalization, Jiangsu Open University, Nanjing 210036, China; 2020204037@stu.njau.edu.cn (B.Y.); lilei@jsou.edu.cn (L.L.); shenmin@jsou.edu.cn (M.S.); 2 College of Horticulture, Nanjing Agricultural University, Nanjing 210095, China; zi_su1@163.com (Y.X.); 2020104081@stu.njau.edu.cn (X.L.)

**Keywords:** tea, secondary metabolites, photosynthetic, antioxidant, overexpression, redundant

## Abstract

Soil acidification in tea (*Camellia sinensis* L.) gardens leads to nutrient depletion, inhibits the growth of tea plants, reduces tea quality, and activates heavy metals such as cadmium (Cd) in the soil. To clarify the impact of soil pH under acidified conditions on tea plant growth physiology and the key genes involved in Cd^2+^ transport in tea plants, this study planted ‘Longjing 43’ under different pH levels (4.0, 4.5, and 5.5) and Cd concentrations (T1 = 0 mg L^−1^, T2 = 0.01 mg L^−1^, T3 = 0.05 mg L^−1^, and T4 = 0.2 mg L^−1^). The results showed that the concentration of Cd in tea plants from highest to lowest was root > stem > mature leaves > young leaves. Under T4, with decreasing pH, the total chlorophyll significantly decreased, the *Fv*/*Fm* significantly decreased, stomatal aperture reduced, and net photosynthetic rate and transpiration rate significantly decreased. In the T4 treatment at pH = 4.0, the contents of free proline and malondialdehyde were both the highest, while superoxide dismutase (SOD), peroxidase (POD), and catalase from micrococcus lysodeiktic (CAT) showed a significant negative correlation with pH. By screening the tea genome data, a total of nine CsHMAs involved in metal ion transport were identified. The qRT-PCR results indicated that the expression level of *CsHMA2* was the highest in young leaves of tea, and *CsHMA2* was localized on the cell membrane. Under T4 and pH = 4.0, transient overexpression of *CsHMA2* enhanced the ability of tea to transport Cd^2+^, whereas transient silencing of *CsHMA2* weakened this ability. These findings not only help understand how tea adapts and regulates its physiological processes in acidic environments but also provide an important theoretical basis and technical guidance for soil improvement in tea gardens, the control of heavy metal pollution, and the optimization of tea quality.

## 1. Introduction

Tea (*Camellia sinensis* L.) is an important economic crop suitable for cultivation in acidic environments; however, excessively acidic soil conditions can inhibit the metabolic activities of tea plants and degrade the quality of tea leaves [1]. With the rapid development of industry, there is an exacerbation of the acidification of tea garden soils due to acid deposition [2]. To avoid the accumulation of heavy metals in tea leaves, it is imperative to address the problem of soil acidification in tea gardens [3]. The main causes of soil acidification in Chinese tea plantations include atmospheric acidification, fertilization methods, planting management methods, and physiological metabolism of tea [4]. There are many reasons for the acidification of tea garden soils, which can be mainly divided into two forms: exogenous acids and endogenous acids [5]. Exogenous acids include acid rain, the return of fallen branches and leaves and pruned branches to the field, and regular fertilization during tea garden management [6]. Endogenous acids consist of hydrogen ion secretion from the root systems of tea plants, as well as organic acids such as oxalic acid, citric acid, and malic acid. The process of increasing H^+^ in the soil is the essence of soil acidification [7].

Soil acidification in tea gardens leads to nutrient depletion, which inhibits the growth of tea plants and reduces the content of secondary metabolites [8]. The content of secondary metabolites in tea plants primarily comprises substances such as tea polyphenols (TP), catechins, caffeine (CAF), amino acids (AA), flavonoids, pigments, and terpenoids [9]. A decrease in available nitrogen in the soil affects the accumulation of AA in tea leaves, although there has been no direct research proving that soil acidification leads to a reduction in the AA content in tea leaves [10]. Catechins are a series of compounds derived from carbon elements, and soil acidification results in the leaching of organic matter in the soil, affecting the types and quantities of microorganisms in the soil and inhibiting the synthesis of catechins in tea [11]. Tea leaves are rich in caffeine, far exceeding other plants [12]. The content of caffeine in tea leaves is closely related to the nitrogen cycle in tea leaves; soil acidification reduces the absorption of carbon and nitrogen content from the soil by tea, thereby decreasing the synthesis of CAF in tea [13]. Soluble sugars (SS) in tea leaves are the main carbohydrates, primarily consisting of glucose, fructose, galactose, and arabinose [14]. Soil acidification alters the form of mineral elements absorbed by tea, thus affecting the synthesis of soluble sugars within the tea. Changes in environmental conditions due to soil acidification impact the absorption of organic matter and mineral elements by tea, influencing the composition and content of major quality components in tea leaves [15].

Soil acidification leads to an increase in the availability of heavy metals in the soil [16]. Heavy metals accumulate in tea leaves through bioaccumulation, posing a toxic threat and severely endangering the natural ecological environment [17]. Cd is one of the most common. Once Cd enters the soil, it is readily captured by the root systems of tea and is transported to the aerial parts where it accumulates, potentially entering the human body through the food chain and posing a potential risk to human health [18]. When the accumulation of Cd in tea plants exceeds the tolerance level, basic physiological functions, including photosynthesis, transpiration, metabolic activities, and antioxidant defense mechanisms, are negatively affected, hindering normal growth and resulting in decreased yield and quality of tea leaves [19]. However, the impact of soil acidification in tea gardens on the absorption of Cd by tea plants remains unclear, and the genes related to the absorption and transport of Cd ions in tea plants have yet to be discovered.

Plants possess regulatory systems for maintaining the balance of metal ion concentrations within their bodies, with heavy metal transport proteins being a crucial component of these systems [20]. Heavy metal transport proteins can be broadly classified into two categories: uptake proteins and efflux proteins. Among them, the main uptake proteins include the Yellow Stripe-Like (*YSL*) transporter family, the Zinc/Iron Regulator (*ZRT/IRT*)-like protein (*ZIP*) family, and the Natural-Resistance-Associated Macrophage Protein (*NRAMP*) family [21]. Efflux proteins include P_1B_-type ATPases and Anion Diffusion Facilitator (*ADF*) protein families, which serve to expel heavy metals from the cytoplasm or transport them to vacuoles [22]. The P_1B_-ATPases are a widely present class of proteins in living organisms that can transport heavy metal ions across membranes via ATP hydrolysis, forming a subclass of the P-type ATPase family. Research has found that P_1B_-ATPases primarily handle the active transport of heavy metal ions in plants and play a vital role in the regulation of heavy metal homeostasis [23]. P_1B_-ATPase genes are widespread in plants, and studies have shown that members of the HMA gene family play significant roles in regulating the uptake and transport of Cd. For example, *OsHMA2* is expressed in the roots and nodes of rice, and the protein is located in the plasma membrane of the root sheath. *AtHMA3* locates in the vacuole membrane and mediates the transport of Zn^2+^ and Cd^2+^ into the vacuole for detoxification [24]. However, there are no reports on the identification and functional studies of HMA gene family members in tea.

To investigate the effects of Cd treatment under acidified soil conditions on the growth physiology of tea plants and the absorption mechanism of Cd under these conditions, this study set up an experiment planting tea with different Cd concentrations under acidified conditions. The experiment measured the Cd concentration in tea, growth indicators, photosynthetic system parameters, and antioxidant enzyme activities. These results are crucial for developing soil amendments for acidified soils; exploring efficient and environmentally friendly application techniques to adjust the pH value of soils; and improving the availability of mineral elements, which is of great significance for the healthy development of soil.

## 2. Results

### 2.1. Effect of pH Value and Cd Treatment on Cd Content in Tea

**Figure 1:**
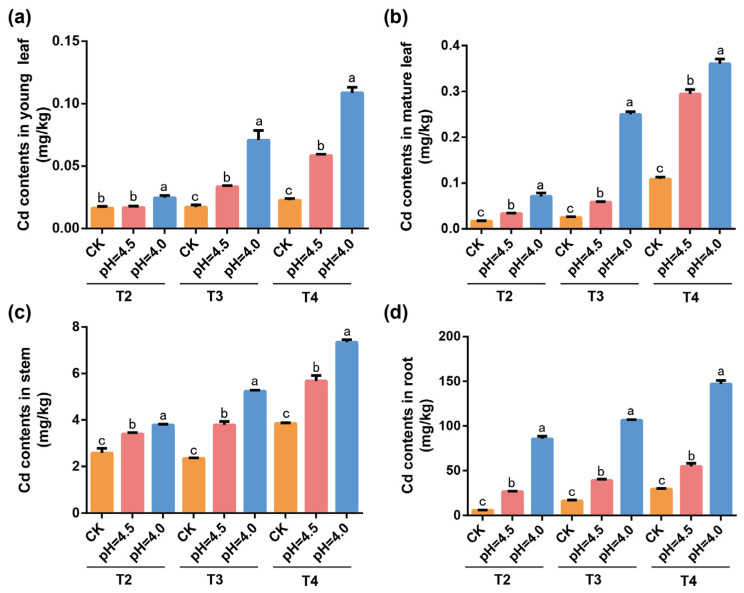
Cd content in different parts of the tea plant under different pH and different Cd concentrations. Note: (**a**) Cd content in young leaves; (**b**) Cd content in mature leaves; (**c**) Cd content in stems; (**d**) the amount of Cd in the root. Different letters indicate statistically significant differences.

The Cd content in the young leaves, mature leave, stems, and roots of tea plants was measured after 7 days under treatment with Cd concentrations (0, 0.05, 0.1, 0.2 mg L^−1^) at pH levels of 5.5, 4.5, and 4.0. The results indicate that the order of Cd concentration in tea from highest to lowest is root > stem > mature leaf > young leaf. Under high-concentration Cd treatment at the same pH value, the Cd content in tea was significantly higher compared to other Cd concentration treatments. The Cd treatment significantly increased the Cd content in all tissues of tea, and the Cd content increased significantly with decreasing pH values. Under the same pH, the Cd content in T4 was significantly higher than in other treatments across all tissues (Figure 1).

### 2.2. Effects of pH Value and Cd Treatment on Secondary Metabolites of Tea

The effects of different pH levels and Cd treatments on secondary metabolites in tea, including AA, TP, CAF, and SS, were measured. As shown in Figure S1, with the continuous decrease in pH, the contents of AA and TP continuously decreased; as the Cd concentration treatment increased, the contents of AA and TP significantly decreased. With the continuous decrease in pH, there was no significant change in the contents of CAF and SS; however, as the Cd concentration treatment increased, the contents of CAF and SS significantly decreased. Notably, under T4 at pH 4.0, the AA content was reduced by 55% compared to T1 under CK. The impacts of different pH levels and Cd treatments on six types of catechins in tea—epigallocatechin (EGC), epicatechin (EC), epigallocatechin gallate (EGCG), epicatechin gallate (ECG), gallocatechin gallate (GCG), and gallocatechin (GC)—were also examined. With the continuous decrease in pH, the contents of GC, EC, and EGCG continuously decreased, and as the Cd concentration treatment increased, the contents of GC, EC, and EGCG significantly decreased. With the continuous decrease in pH, there was no significant change in the contents of ECG, GCG, and GC; similarly, as the Cd concentration treatment increased, these three catechins showed no significant change. Among them, under T4 at pH 4.0, the EC content was approximately three times lower than that of T1 under CK.

### 2.3. Effects of pH Value and Cd Treatment on Phenotypes of Tea

As shown in Figure S2, in the CK, the growth of tea buds and roots was inhibited, but the degree of inhibition did not significantly increase with increasing Cd concentration and treatment time. In the group with pH 4.0, the growth of tea buds and roots was markedly inhibited, and the degree of inhibition became more pronounced with increasing Cd concentration and treatment time. By day 21, T4 at pH 4.0 had a significant inhibitory effect on the elongation of tea buds, the elongation of new roots, and the number of new root tips. In T1 to T4 shown in Figure S3, the elongation of buds significantly decreased as the pH decreased. At the same pH level, the elongation of buds progressively decreased as the Cd concentration increased. The trend in the elongation of root tips was similar to that of bud elongation, but the significance was less pronounced.

### 2.4. Effects of pH Value and Cd Treatment on Photosynthetic System Indexes of Tea

**Figure 2:**
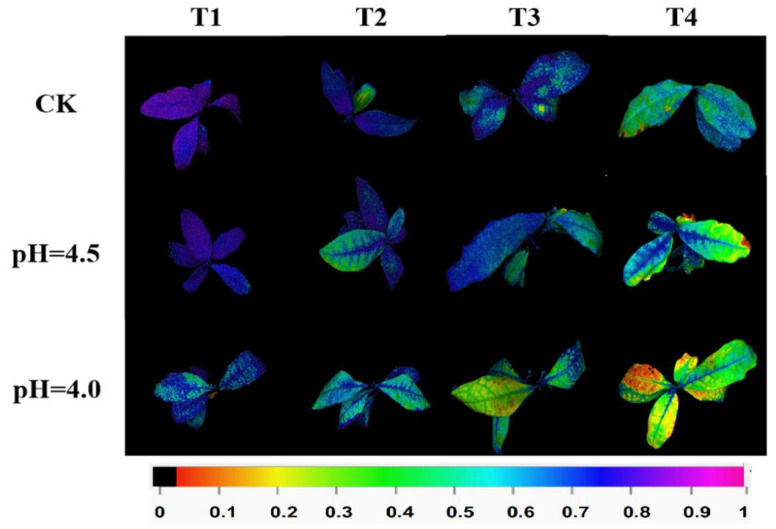
Pseudo color image of *Fv*/*Fm* under different pH and Cd concentrations. Purple-blue color indicates normal state of the photosynthetic apparatus, while green and yellow colors indicate damage to photosystem II caused by different pH and Cd concentrations.

The chlorophyll fluorescence in vivo of tea seedlings was observed under different pH levels and Cd concentrations. Purple-blue indicates that the photosynthetic organs are in a normal state, while green, yellow, and red indicate damage to Photosystem II due to Cd stress. Damage to plants worsened with increasing Cd concentration across all pH treatments. Similarly, damage worsened with decreasing pH levels across all Cd concentration treatments. The most severe damage occurred under T4 at pH 4.0 (Figure 2). As illustrated in Figure S4a, the phenotypic effects of different pH levels and Cd concentrations on tea were observed. In the CK, T4 showed a phenotype of red threads and wilting compared to T1-T3. At pH 4.0, T2-T4 exhibited yellowing, red threads, and wilting compared to T1. Under T1, there was no significant change in leaf phenotype with decreasing pH. However, under T4, the leaf phenotype showed significant yellowing and wilting with decreasing pH, along with a marked increase in red threads. The effects of different pH levels and Cd treatments on chlorophyll a, chlorophyll b, carotenoids, and total chlorophyll content were also examined. In Figure S4b, it was found that within each group, chlorophyll a content continuously decreased with increasing Cd concentration. In the CK, the chlorophyll a content in T4 and T3 was significantly lower than in T1 and T2. At pH 4.0, the chlorophyll a content in T4 was significantly lower than in other treatments. As shown in Figure S4, the trends in chlorophyll b, carotenoids, and total chlorophyll content across different pH levels and Cd treatments were consistent with the trend in chlorophyll a content. Under different pH levels and Cd treatments, the *Fv/Fm* ratio continuously decreased with increasing Cd concentration. It also continuously decreased with decreasing pH levels. The *Fv/Fm* ratio reached its lowest point under T4 and pH 4.0 (as shown in Figure S5).

**Figure 3:**
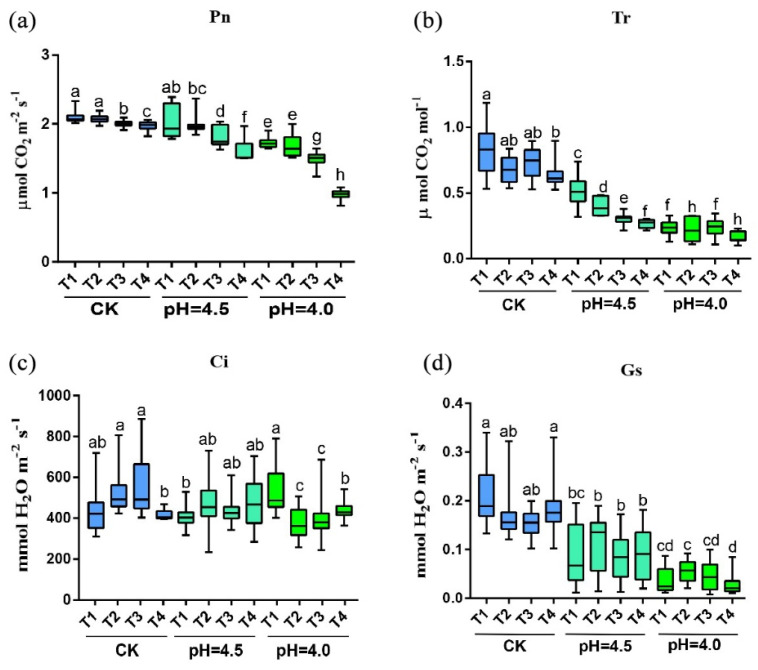
Effects of different pH and Cd concentrations on photosynthetic parameters of tea leaves. Note: (**a**) net photosynthetic rate; (**b**) transpiration rate; (**c**) intercellular carbon dioxide; (**d**) stoma conductance. Different letters indicate statistically significant differences (*p* < 0.05).

Photosynthesis is the most fundamental life activity for plant growth and metabolism. This study measured the photosynthetic parameters of tea after treatment with different pH levels and Cd concentrations. The results are shown in Figure 3. As the duration of stress treatment increased, the transpiration rate (Tr), net photosynthetic rate (Pn), and stomatal conductance (Gs) of tea plant leaves all showed a gradual downward trend, while the intercellular CO_2_ concentration (Ci) did not change significantly during the stress period. Among these, the Tr, Pn, and Gs parameters in the CK at the tested Cd concentration were significantly higher than those in the pH = 4.0. It is noteworthy that under high Cd concentration treatment, the Pn and Tr parameters of the tea in both the CK and the pH = 4.0 showed a significant decrease, indicating that acidification exacerbated the photosynthetic and respiratory stresses on the tea leaves caused by Cd. The reduction in the photosynthetic rate of tea leaves was due to non-stomatal factors such as the weakening of mesophyll photosynthetic capacity. As shown in Figure S6, the state of stomata was observed under a focal length of 100 μm, and the degree of stomatal opening and closing was recorded. The effect of stomatal opening and closing is reflected by the ratio of stomatal length to width. Under T1–T4 in the CK, there was no significant difference in the degree of stomatal opening and closing; under T1–T4 at pH = 4.5, a significant difference in the degree of stomatal opening and closing was observed; and under T1–T4 at pH = 4.0, an extremely significant difference in the degree of stomatal opening and closing was noted (Figure S7).

### 2.5. Effects of pH Value and Cd Treatment on the Antioxidant System of Tea

**Figure 4:**
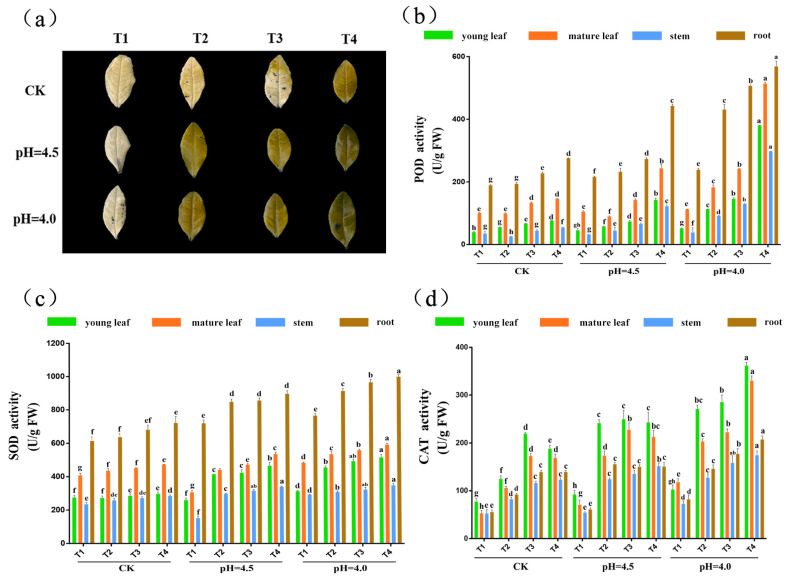
Effects of different pH and Cd concentrations on the enzyme activity of tea plants. Note: (**a**) H_2_O_2_ histochemical staining; (**b**) SOD activity; (**c**) POD activity; (**d**) CAT activity. Different letters indicate statistically significant differences (*p* < 0.05).

Figure 4a shows the results of H_2_O_2_ histochemical staining of tea leaves treated with NBT solution, where dark blue spots indicate the accumulation of H_2_O_2_. A lower degree of decolorization suggests more severe stress on the leaves. It can be observed that the tea leaves subjected to different pH and Cd treatments exhibited varying degrees of dark blue spots and decolorization. Compared to T1 in the CK, T4 in the CK almost failed to decolorize, with few dark blue spots. T1 at pH 4.0 completely decolorized, with an increase in dark blue spots. T4 at pH = 4.0 not only failed to decolorize but also showed large areas of dark blue spots.

As shown in Figure 4, enzymes related to plant antioxidant defense include SOD, POD, and CAT. When plants suffer from abiotic stress, a large amount of free oxygen accumulates in the body, and the antioxidant enzyme system is induced to remove oxygen free radicals. The activity levels of SOD, POD, and CAT indicate the impact of Cd on the antioxidant enzymes in tea. Overall, the activity levels of SOD and POD were root > mature leaf > young leaf > stem. Under the same pH conditions, the SOD activity in the roots increased with the increase in Cd content; under the same Cd content treatment, the SOD activity significantly increased with the decrease in pH. There was no significant change in SOD activity in young leaves, mature leaves, and stems in the CK when the Cd content changed; however, in the pH 4.5 and pH 4.0, the SOD activity significantly increased with the increase in Cd content. The trend in POD activity in the roots was consistent with that of SOD. In young leaves, mature leaves, and stems, the POD activity significantly increased only in the pH 4.0 with the increase in Cd content. Overall, the CAT activity levels were young leaf > mature leaf > root > stem. Under the same pH conditions, the CAT activity in young leaves and mature leaves increased with the increase in Cd content; under the same Cd content treatment, the CAT activity significantly increased with the decrease in pH. In the pH 4.0 group, the POD activity in all tissues of tea plants increased with the increase in Cd content.

As shown in Figure S8a, different pH levels and Cd concentrations affected the content of free proline in tea leaves, with the order being: mature leaf > young leaf > root > stem. Under the same pH conditions, the content of free proline in young leaves, mature leaves, and roots increased with the increase in Cd concentration, while there was no significant change in the stem. Under T4 at pH 5.5, pH 4.5, and pH 4.0, the content of free proline in young leaves, mature, and roots was higher than in other treatments, with T4 at pH 4.0 having the highest content, which was significantly higher than other treatments (*p* < 0.05). The content of free proline under T4 and pH 4.5 was higher than under T3 and pH 4.0. The malondialdehyde (MDA) content in tea plant leaves under different pH and Cd treatments was measured, with the order being young leaf > mature leaf > stem > root, as shown in Figure S8b. Under the same pH conditions, the MDA content in young leaves increased with the increase in Cd concentration. In T4 under the three different pH values, the MDA content was higher than in other treatments, with T4 and pH 4.0 having the highest MDA content.

### 2.6. Analysis of the Expression Pattern of the CsHMAs Gene

Through the screening of tea plant genome data, we identified a total of nine *CsHMA* (Heavy Metal ATPase) genes that are responsible for transporting metal ions. They are named *CsHMA1*-*CsHMA9* (Table S1). To elucidate the evolutionary relationships of these HMA genes in tea plants, we performed a phylogenetic analysis comparing HMAs from tea plants, Arabidopsis thaliana, rice, kiwifruit, and grape (Figure S9). The analysis revealed that the tea plant HMA genes grouped into distinct clusters: *CsHMA2* forms its own clade; *CsHMA1* and *CsHMA3*, as well as *CsHMA6* and *CsHMA8*, cluster together in one clade; *CsHMA7* forms a separate clade; *CsHMA9* forms another distinct clade; and *CsHMA4* and *CsHMA5* cluster together in their own clade. Members of the tea plant HMA gene family can be classified into two groups based on their metal substrate specificity: Zn^2+^/Co^2+^/Cd^2+^/Pb^2+^ P_1B_-ATPase (Zn sub-group) and Cu^2+^/Ag^2+^ P_1B_-ATPase (Cu sub-group). Phylogenetic analysis, combined with comparisons to previous studies, indicates that the *CsHMA1*, *CsHMA2*, and *CsHMA3* genes in tea plants belong to the Zn^2+^/Co^2+^/Cd^2+^/Pb^2+^ P_1B_-ATPase, while the remaining genes are classified under the Cu^+^/Ag^+^ P_1B_-ATPase sub-group.

**Figure 5:**
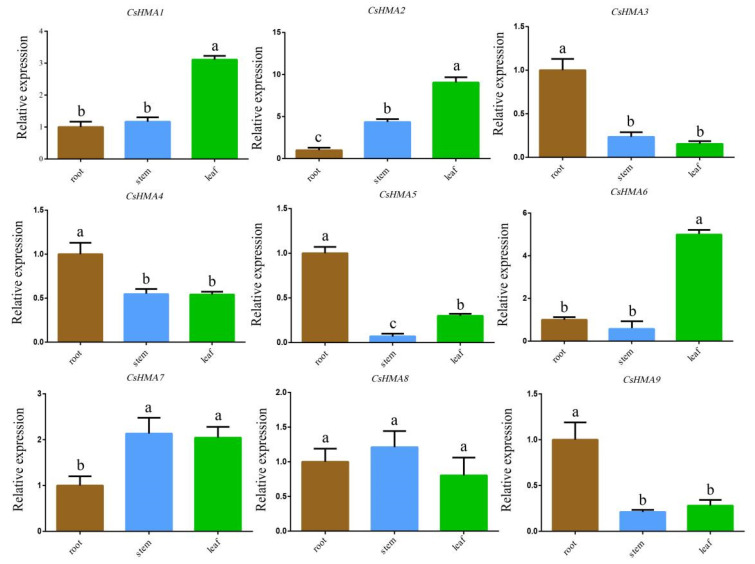
Expression of *CsHMA* genes of *Camellia sinensis* in different tissues (root, stem, and leaf) of the tea. Note: All data were the mean ± standard deviation of 3 biological replicates, each containing 3 technical replicates. Different letters indicate statistically significant differences (*p* < 0.05).

The expression levels of nine *CsHMAs* genes in the roots, stems, and leaves of tea were analyzed using qRT-PCR. The *CsHMAs* genes were expressed to varying degrees in different tissues of the tea. As shown in Figure 5, *CsHMA1*, *CsHMA2*, and *CsHMA6* were primarily expressed in the leaves, while *CsHMA3*, *CsHMA4*, *CsHMA5*, and *CsHMA9* were mainly expressed in the roots. *CsHMA7* and *CsHMA8* were predominantly expressed in the stems, with *CsHMA2* also showing high expression in the stems. Genes belonging to the Zn subfamily exhibited similar expression characteristics, with the highest expression in the leaves. Genes of the Cu subfamily showed higher expression in the above-ground parts, specifically in the stems and leaves.

**Figure 6:**
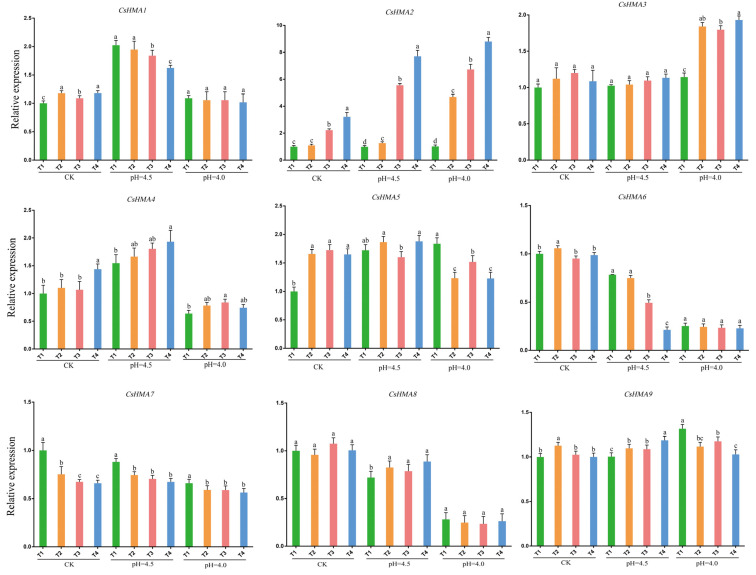
The expression patterns of *CsHMA* genes in leaves of tea plants under different pH and Cd concentrations. Note: All data were the mean ± standard deviation of 3 biological replicates, each containing 3 technical replicates. Different letters indicate statistically significant differences (*p* < 0.05).

As shown in Figure 6, the expression patterns of *CsHMAs* genes in tea plant leaves varied under different pH levels and Cd concentrations. The expression pattern of *CsHMA2* was the most prominent. When pH = 5.5, the gene expression increased with the rise in Cd concentration. Under the same Cd concentration, the gene expression also increased as the pH decreased. The lower the acidity and the higher the Cd concentration, the more the expression of *CsHMA2* was stimulated, reaching the highest expression under highly acidic and high Cd concentration conditions. The expression pattern of *CsHMA4* was similar to that of *CsHMA2* only under CK and pH 4.5.

The expression patterns of *CsHMA* genes in stems also differed under various pH levels and Cd concentrations. *CsHMA1*, *CsHMA3*, and *CsHMA9* showed different patterns of increased expression under pH and Cd. The expression of *CsHMA1* was more affected by the decrease in pH, while that of *CsHMA3* and *CsHMA9* was more influenced by changes in Cd concentration. When pH = 5.5, the pH decreased, and the expression levels of *CsHMA2* increased significantly under T2, T3, and T4, with no significant change under T1. As the pH decreased, the expression of *CsHMA2* increased significantly with the increase in Cd concentration. Other members of the *CsHMAs* family also showed varying degrees of increased or decreased expression under different treatments, but the patterns were not clear (Figure S10).

Figure S11 shows the expression patterns of *CsHMA* genes in tea plant roots under different pH and Cd concentrations. It was found that at pH 5.5 and pH 4.5, the expression of *CsHMA1* was high under high-concentration Cd treatment (T4), while at pH 4.0, the expression was highest under medium-concentration Cd treatment (T3). Under T4, the expression of *CsHMA2* and *CsHMA3* increased continuously as the pH decreased, and as the Cd concentration increased, the expression of both genes rose, reaching the highest expression under T4 and pH 4.0. The expression of *CsHMA6* was highest under T3, while the expression of *CsHMA7* was highest under T4, with the expression trend of this gene being independent of pH changes. The expression of *CsHMA8* decreased under the combined stress of Cd and low pH, while the expression trends of other genes were not significant under the treatments.

### 2.7. Transient Overexpression of CsHMA2 Decreased Cd Tolerance in Tea

To determine the subcellular localization of *CsHMA2* in plant cells, a GFP-tagged plant expression vector pBI121-*CsHMA2* (35S::CsHMA2-GFP) was constructed. Using Agrobacterium-mediated transient transformation, this construct was overexpressed in transgenic tobacco leaf cells containing a nuclear marker. An empty vector pBI121-*CsHMA2* (35S::GFP) without the *CsHMA2* protein served as a negative control. The results showed that in tobacco leaf cells transiently overexpressing 35S::GFP, green fluorescence signals were detected in both the nucleus and the plasma membrane. In contrast, in cells expressing 35S::*CsHMA2*-GFP, green fluorescence signals were only observed at the plasma membrane (Figure S12), indicating that *CsHMA2* is localized to the plasma membrane.

Quantitative analysis of transient overexpression lines and no-load lines showed that the *CsHMA2* expression level in transient overexpression lines was significantly higher than that in no-load lines (Figure S13a). Under Cd stress, *CsHMA2*-overexpressing lines showed more serious symptoms of root blackening, bud wilting, and brown change in young leaves than unloaded lines (Figure S13e). In terms of the in vivo fluorescence phenotype, it was found that compared with the blue-violet fluorescence of the no-load strain, the *CsHMA2*-overexpressed strain showed yellow-green fluorescence and a little red fluorescence, indicating that the overexpressed strain was more severely stressed (Figure S13f). The results of Cd content determination showed that Cd content in stems and leaves of *CsHMA2*-overexpressing lines under Cd stress was significantly higher than that in no-load lines, while there was little difference in Cd content in roots (Figure S13b).

### 2.8. Transient Interference with CsHMA2 Increased Cd Tolerance in Tea

Quantitative analysis of instantaneous interference lines and no-load lines showed that the expression of *CsHMA2* in no-load lines was significantly higher than that in instantaneous silence lines of *CsHMA2* (Figure S14a). Under Cd stress, there was no significant difference between the transient interference of *CsHMA2* and the phenotype of unloaded plants, but in terms of in vivo fluorescence phenotype, the purple proportion of blue-violet light of silent plants was higher than that of unloaded plants, indicating that the plants were less stressed (Figure S14e,f). The results of Cd content determination showed that under Cd stress, the Cd content in the leaves of *CsHMA2* transient interference lines was significantly lower than that of no-load lines, and there was no significant difference in the Cd content in the roots of the two lines (Figure S14b).

### 2.9. Transient Overexpression of CsHMA2 in Acidified High-Concentration Cd Combined Treatment

**Figure 7:**
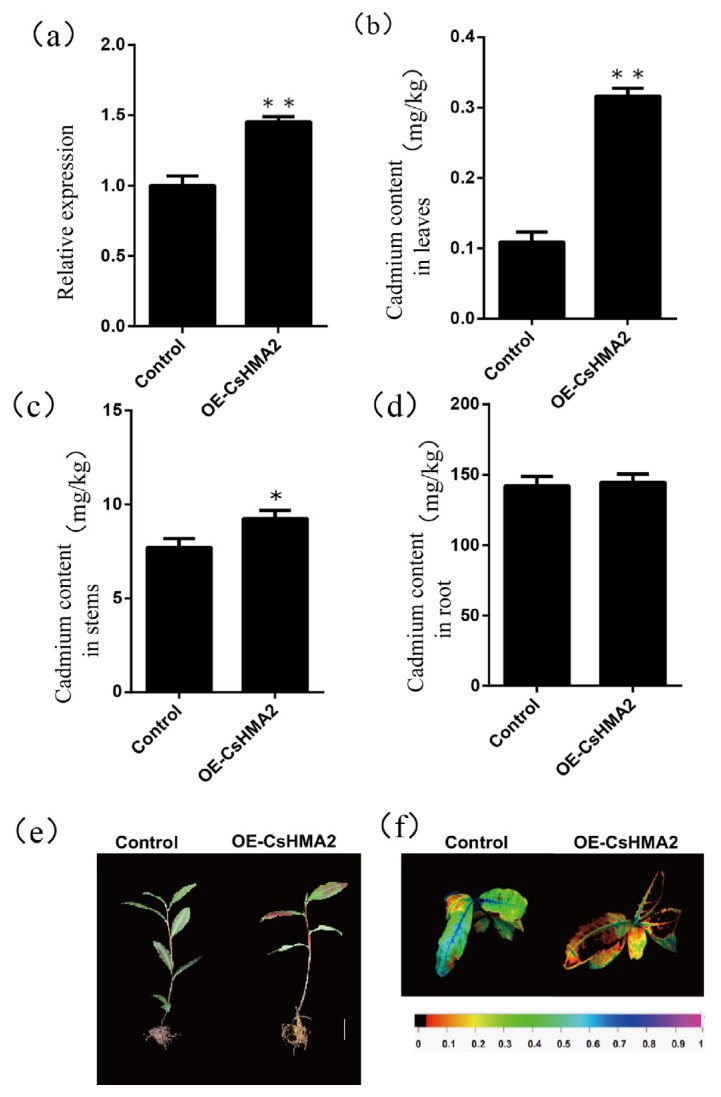
The *CsHMA2* gene was overexpressed at a high concentration of Cd under acidification. Note: (**a**) *CsHMA2* gene expression; (**b**) Cd content in leaves; (**c**) Cd content in stems; (**d**) Cd content in roots; (**e**) tea plant phenotype; (**f**) live fluorescence of tea plant. * *p* < 0.05, ** *p* < 0.01; the scale in the figure represents 1 cm.

Quantitative analysis revealed that under high-concentration Cd stress in acidic conditions, the expression level of the *CsHMA2* gene in overexpressing lines was significantly higher than that in the empty vector lines (Figure 7a). Under the combined stress of acidification and Cd, compared to the empty vector lines, the *CsHMA2*-overexpressing plants exhibited phenotypes of wilting and reddening (Figure 7e). This phenotype was more severe in terms of browning, wilting, and leaf shedding compared to plants subjected to Cd stress alone. Live fluorescence also showed consistent results (Figure 7f). This indicates that the dual stress exacerbates the damage to the plants. The results of Cd content measurement in *CsHMA2*-overexpressing plants under dual stress showed that, under dual stress treatment, the Cd content in the stems and leaves of the *CsHMA2*-overexpressing lines was significantly higher than that in the control empty vector lines, while the difference in Cd content in the roots between the two was not significant (Figure 7b).

### 2.10. Transient Interference of CsHMA2 by Acidizing High-Concentration Cd Combined Treatment

As shown in Figures S15, under the combined stress of acidification and Cd, the symptoms in the *CsHMA2*-silenced lines were significantly milder compared to the empty vector lines, with only slight red spots on the young leaves (Figure S15e). Compared to the single Cd stress treatment, the symptoms in the *CsHMA2*-silenced lines were more severe under dual stress treatment. The results of live fluorescence were consistent with the phenotypic observations (Figure S15f). The Cd content measurements showed that, compared to the single Cd stress treatment, the Cd content in the leaves of the *CsHMA2*-silenced lines increased but remained significantly lower than in the empty vector lines. The Cd content in the stems and roots did not differ significantly from that under the single Cd stress treatment (Figure S15b). These results indicate that acidification can exacerbate the damage caused by Cd stress in tea.

## 3. Discussion

In this study, under the treatment of pH 4.0 and a Cd concentration of 0.2 mg kg^−1^, the roots of tea directly absorbed and immobilized a large amount of Cd, leading to increased Cd content in all tissues of the tea. Compared to the CK, the Cd content in all tissues of tea treated with low pH and high Cd concentration was significantly higher. The Cd content increased in the order of root > stem > mature leaf > young leaf, which is similar to the pattern of Cd absorption in other plants [25]. The uptake of Cd by plants mainly comes from the soil, and there is a mechanism for accumulating Cd in the roots and stems of tea. In the results of this study, Cd elements showed a significant negative correlation with secondary metabolites in tea leaves. As the Cd content in various tissues of tea increased, the levels of secondary metabolites decreased, with the most significant reductions observed in AA and TP. The reduction in AA can be attributed to two factors: one is the decrease in pH, which leads to the transformation of NH_4_^+^ into nitrate nitrogen, affecting the absorption of nitrogen in the nutrient solution [26], the other is the impact of increased Cd absorption on the formation of AA in tea [23]. The reduction in TP is mainly due to the decrease in the content of EGC, EC, and EGCG, with EGCG being the most abundant among catechins and a precursor for the formation of simple catechins. EC plays a crucial role in the pleasant taste of simple catechins in tea leaves. The increase in Cd reduces the content of CAF, thereby degrading the quality of tea [27].

Soil acidification can inhibit the growth and development of plants. In this study, tea treated with pH 4.0 showed slow growth, with a reduction in the elongation of buds, the number of new root tips, and the elongation of roots. This is consistent with the results of Lin et al., which further proves that soil acidification can inhibit plant growth and development [28]. Under the influence of acidic environment, the growth of tea was significantly slowed down, and when the concentration of Cd was too high, the growth and development of tea was significantly inhibited, which was consistent with the results of Huybrechts’ study that Cd had toxic effects on the growth and development of tea [29]. Our results showed that an acidic environment exacerbated the effect of low Cd concentration on tea growth and development, and it accelerated the growth and development effect caused by the enhancement of tea Cd absorption ability, which was consistent with the results of Ju et al. [30]. Compared to the control, both pH 4.5 and pH 4.0 conditions reduced bud numbers, indicating that acidity and Cd concentration inhibit bud development. High Cd concentrations also significantly decreased root tip elongation and the number of root tips, suggesting that Cd^2+^ imposes greater phenotypic stress on roots than on buds and stems, consistent with findings in rice studies [31]. Under high-concentration Cd treatment, the content of chlorophyll was significantly inhibited, with the lowest chlorophyll content in tea leaves observed under T4 and pH 4.0. This may have been due to the fact that Cd stress causes key enzymes in the chlorophyll synthesis pathway, such as protochlorophyllide reductase and porphobilinogen deaminase, to undergo conformational changes, thereby reducing their activity and decreasing chlorophyll synthesis [32]. Additionally, acidification activates Cd^2+^, and active Cd hinders the absorption of trace elements required for chlorophyll synthesis and disrupts the integrity of the membrane structure where chlorophyll is located, leading to the disintegration of the grana stacking structure within chloroplasts and causing irreversible damage to chloroplasts [33]. Despite no significant decrease in chlorophyll content with decreasing pH, increasing Cd concentrations led to a decline in chlorophyll levels. This suggests that Cd stress affects chlorophyll synthesis and adaptation mechanisms in tea plants [34]. Heavy metals inhibit photosynthesis by affecting the electron transport process in photosynthetic reactions and disrupting the structure of chloroplasts, thus damaging the integrity of the photosynthetic system [35]. In this study, compared to T1, the stomatal conductance, Pn, and Tr of T4 at pH 4.0 were significantly reduced. As the Cd concentration increased, the stomata of the leaves may close, reducing transpiration and leading to a decrease in photosynthesis [36].

The content of free proline in leaves and roots increased with the increase in Cd concentration under pH 4.5 and pH 4.0 treatments, indicating significant accumulation of proline, which suggests that tea is under heavy metal Cd stress in an acidic environment. Therefore, by measuring MDA, we can indirectly assess the extent of lipid peroxidation and the damage to the membrane system, as well as the plant’s stress resistance. Membrane and cell damage can lead to MDA accumulation, primarily by inducing lipid peroxidation, damaging the structure of biological membranes, especially the plasma membrane, leading to structural and functional damage to cell membranes, altering membrane permeability, and thus affecting the normal progression of various physiological and biochemical reactions [37]. The MDA is the final decomposition product of lipid peroxidation in membranes and reacts with proteins, causing them to lose function. The content of MDA can reflect the degree of damage to plants under stress [38]. In this study, under Cd stress conditions, the antioxidant enzyme system in tea was significantly activated to cope with the oxidative stress brought by Cd. The content of SOD increased only with the increase in Cd concentration under pH 4.0. However, the content of POD and SOD significantly increased with the increase in Cd concentration under pH 4.5 and pH 4.0. Combining the analysis of superoxide anion content through NBT staining, it was found that tea cells under high-concentration Cd treatments at pH 4.5 and pH 4.0 were severely stressed. POD and CAT were activated to assist in the conversion of superoxide anions into water during this process [39].

Based on the specific expression of *CsHMAs* genes in different tissues of tea plants and under various pH and Cd treatments, we infer the role of these metal ion transporters in the transport of metal within tea. *CsHMA2* and *CsHMA3* all belong to the Zn subfamily but have distinct expression patterns. The expression of the *CsHMA2* is significantly higher in leaves and stems than in roots, suggesting that *CsHMA2* may have a role similar to the transport of Zn^2+^ and Cd^2+^, and its expression is relatively high during transport in stems and leaves. *CsHMA3* is highly expressed only in roots, which is similar to the results in Arabidopsis, rice, and tobacco, where it is primarily localized in the vacuolar membrane of roots [40]. This differs from the transport of Zn^2+^ by the *AtHMA4* gene in Arabidopsis and the *AhHMA4* gene in barley, which may be a specific expression of the tea gene [41]. Previous studies have shown that *HMA2* and *HMA4* genes in different crops are key genes for the transport of Cd^2+^ [42]. In the T1 treatment of roots, stems, and leaves, changes in pH did not significantly affect the expression of *CsHMA2*, indicating that single acidification changes cannot regulate the expression of *CsHMA2*. Under the same pH treatment, as the Cd concentration increased, the expression of the *CsHMA2* gene also increased, suggesting that *CsHMA2* responds directly or indirectly to Cd stress. Under the same Cd concentration treatment, the lower the pH, the higher the expression of *CsHMA2*, which may be due to the increased activation of Cd^2+^ as the acidity decreases, leading to the absorption of more Cd^2+^ by the tea. Therefore, we infer that *CsHMA2* is a key gene responsible for the transport of Cd ions in tea plants and that its function is influenced by pH. Zhao et al. found that *OsHMA2* in rice serves as a crucial transporter for cadmium (Cd). *OsHMA2* is expressed in the roots and nodes of rice plants, where it localizes to the plasma membrane of root pericycle cells and the phloem of nodes. Mutation of *OsHMA2* decreases the translocation of Cd from roots to shoots. These observations align with our findings [43].

Overexpression of *CsHMA2* exacerbated Cd stress damage to young leaves, leading to an increase in Cd content in young leaves. Conversely, silencing *CsHMA2* alleviated Cd stress, resulting in a decrease in Cd content in young leaves. This indicates that the accumulation of Cd in young leaves is closely related to the expression level of *CsHMA2*. Under high-concentration Cd stress in acidified conditions, the trends in the overexpression and silencing of *CsHMA2* were similar to those under high-concentration Cd stress alone. The main reason for this could be that the decrease in pH accelerates the activity of heavy metal ions such as Cd^2+^, while simultaneously reducing the activity of metal ions such as Cu^2+^ and Zn^2+^ [44]. The increased expression of *CsHMA2* enhances the transport efficiency of Cd^2+^, thus yielding similar results to those observed under high-concentration Cd stress alone.

## 4. Material and Methods

### 4.1. Materials and Growth Conditions

The tea variety used was the one-year-old ‘Longjing 43’ seedlings. Seedlings with consistent growth were selected and transferred to a light incubator with the following settings: 16 h of light (25 °C), 8 h of darkness (20 °C), light intensity of 300 µmol (m^2^·s)^−1^, and humidity at 75%. ‘Longjing 43’ seedlings were grown at the College of Horticulture, Nanjing Agricultural University.

**Figure 8:**
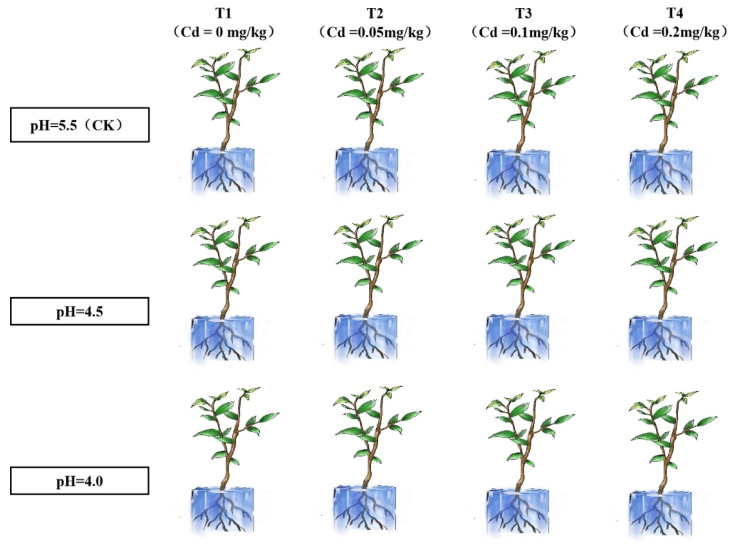
Experimental design diagram of different pH and different Cd concentrations.

As shown in Figure 8, three pH levels were set: pH = 5.5 as the control group (CK), pH = 4.5 as the mildly acidified group, and pH = 4.0 as the acidified group. The pH of the prepared nutrient solutions was adjusted using sodium hydroxide (NaOH) and sulfuric acid (H_2_SO_4_). Four Cd treatments were established: T1 with no added Cd (Cd = 0 mg L^−1^), T2 with low concentration of Cd (Cd = 0.01 mg L^−1^), T3 with normal concentration of Cd (Cd = 0.05 mg L^−1^), and T4 with high concentration of Cd (Cd = 0.2 mg L^−1^). For the Cd concentration treatments, different amounts of CdSO_4_ were added to the full-strength nutrient solution according to the required concentrations. The configuration of nutrient solution follows the paper of Li et al. [45].

### 4.2. Phenotype Observation and Determination of Major Quality Components and Cd Content in Tea

Under varying pH and cadmium treatments, photographs were taken to document the root growth of tea seedlings at days 0, 7, 14, and 21. The number of emerging buds was observed and counted, while the stem diameter was measured using a vernier caliper. Tea seedlings with uniformly developed root systems were selected, washed clean with water, and gently dried. The root systems were then scanned by placing them in a tray filled with 200 mL of distilled water, which was horizontally positioned within the WinRHIZO root analysis system. Once the water had settled, the new roots of the tea seedlings were carefully excised and laid flat on the tray for photography and the measurement of parameters such as root length and root tip count.

On the 7th day of the experiment, we collected one bud and two leaves from the tea plant, along with its stems and roots as experimental materials. One bud and two leaves of the tea seedlings were subjected to microwave fixation and drying for the determination of total free amino acids (AA), tea polyphenols (TP), caffeine (CAF), soluble sugars (SS), and six types of catechins. The determination methods followed national standards [45]. A sample of 0.2 g of the dried tea leaves, stems, and roots was weighed. The Cd content was detected using the ICP-MS standard sample preparation method (Optima 8000, Perkin Elmer, Waltham, MA, USA).

### 4.3. Determination of Photosynthetic System Parameters

On the 7th day of the experiment, fresh leaves of the tea seedlings were taken, the midribs were removed, and the leaves were cut and weighed at 0.2 g. They were then ground to a uniform paste and re-ground with 95% ethanol until the tissue turned white, after which they were left to stand for 5 min. The mixture was filtered into a 25 mL volumetric flask, diluted with ethanol, and mixed thoroughly. Using 95% ethanol as the blank, the absorbance of the extract was measured at wavelengths of 665 nm and 649 nm. The chlorophyll content (mg g^−1^) was calculated as follows: (concentration × extraction volume × dilution factor)/fresh weight of the sample. To measure the chlorophyll fluorescence characteristics, an IMAGING-PAM chlorophyll fluorescence imaging system was used to capture images of the plants on day 0 and day 7 after planting, recording the *Fv/Fm* value, which represents the maximum quantum efficiency of PSII primary photoenergy conversion. For the determination of photosynthetic parameters, the entire plant was placed in a dark environment for more than 30 min, and a portable chlorophyll fluorescence meter was used to measure the second leaf from the top of the tea plant (avoiding the main vein), with at least 15 measurements per treatment on the 7th day of the experiment [45]. A Li-6400XT portable photosynthesis system was used to measure Pn, Gs, Ci, and Tr [46]. At least five measurements were made for each treatment, and the built-in light intensity was set to 600 µmol (m^2^·s)^−1^ [47].

### 4.4. Determination of Antioxidant Enzyme Activities

On the 7th day of the experiment, 0.2 g of fresh leaves from the tea seedlings was ground into a powder in a pre-cooled mortar with liquid nitrogen. Then, 1.8 mL of phosphate buffer (50 mM, pH = 7.2) was added and the mixture was further ground into a homogenate. After centrifugation at 6000 g for 10 min, the supernatant was collected and used for the determination of enzyme activities. Superoxide dismutase (SOD) activity: SOD activity was determined using the xanthine oxidase method (hydroxylamine method). Peroxidase (POD) activity: POD activity was measured using the guaiacol colorimetric method. Catalase (CAT) activity: CAT activity was determined using the ammonium molybdate method. Nitroblue tetrazolium (NBT) staining: On the 7th day of the cd stress treatment, leaf segments about 10 cm long were cut from the tea leaves and immersed in NBT reaction solution (2 mg mL^−1^, pH = 7.8, 50 mmol L^−1^ sodium phosphate buffer) in the dark for 8 h. Subsequently, a decolorizing solution was prepared in the ratio of ethanol/acetic acid/glycerol = 30:10:10. The leaves were decolorized in a boiling water bath at 100 °C until completely green-free, and then the stained leaf tissues were observed and photographed. Free proline content determination: Free proline content was determined using fresh leaves from the 7th day of the experiment. Free proline was extracted from the plant material using sulfosalicylic acid. Under acidic conditions, proline reacts with ninhydrin to form a stable red compound, which was extracted with toluene and the absorbance was measured at 520 nm. The determination of free proline content was carried out according to the experimental method of Yang [48].

MDA content determination: Fresh leaves from the 7th day of the experiment were used to determine MDA content. One gram of tea leaf sample was thoroughly ground in liquid nitrogen, and then 10 mL of 10% trichloroacetic acid (TCA) was added. The mixture was centrifuged at 4000 rpm for 10 min, and the supernatant was collected as the sample extract. Two milliliters of the sample extract were mixed with 2 mL of 0.6% thiobarbituric acid (TBA) solution, and the mixture was immediately placed in a boiling water bath for 15 min. After the water bath, the mixture was rapidly cooled and centrifuged at 4000 rpm for 10 min. The supernatant was then transferred to a microplate, and the absorbance levels were measured at 532 nm, 600 nm, and 450 nm using a multifunctional microplate reader. The MDA concentration was calculated using the following formula:

C = 6.45 × (OD_532_ − OD_600_) − 0.56 × OD_450_

### 4.5. qRT-PCR Analysis of CsHMAs Gene Expression in Different Tissues of Tea

To clarify the expression patterns of the *CsHMAs* genes in different tissues of tea plants, tissue samples including roots, stems, and leaves were collected. Total RNA was extracted according to the instructions provided with the plant RNA extraction kit. cDNA was synthesized from the total RNA using the PrimeScript RT reagent kit according to the manufacturer’s protocol. Quantitative real-time PCR (qRT-PCR) was performed using the TB Green Premix Ex Taq II kit, with the tea plant gene *Csβ-Actin* (Table S1) serving as the internal reference gene [45]. The qRT-PCR analysis was conducted using a quantitative PCR instrument (Bio-Rad, Hercules, CA, USA). The relative expression levels of the genes were calculated based on the threshold cycle (Ct) values of the target genes and the internal reference gene, using the 2^−ΔΔCT^ method [49].

### 4.6. Functional Validation of CsHMAs Genes in Tea

To test whether the *CsHMAs* genes affect the Cd stress tolerance of tea plants, transient overexpression of the gene was verified. Materials: Cloning vector pEASY-Blunt, plant overexpression vector pBI121, and TRV2 and TRV1 vectors for transient interference, all preserved in our laboratory. Bacterial strains: Escherichia coli *DH5α* and Agrobacterium tumefaciens GV3101 (Table S2). Procedure: Prepare the suspension solution with 10 mM MES, 10 mM MgCl_2_, and 150 µM acetosyringone. Activate the Agrobacterium tumefaciens strains carrying the pBI121-GFP and pBI121-*CsHMAs* recombinant plasmids, centrifuge at 8000 rpm for 5 min, discard the supernatant, and resuspend the bacterial pellets in the suspension solution. Mix well by vortexing and adjust the OD_600_ to 0.8–1.0. Incubate in the dark for 4–5 h.

Select tea seedlings of the ‘Longjing 43’ variety with consistent growth. Gently puncture the leaf surface with a needle, then use a 1 mL syringe without a needle to draw up the suspension solution and inject it into the lower epidermis of the leaves, spreading it across the entire leaf. Inject 2–3 leaves per tea plant, with at least three tea seedlings injected. Use tea seedlings injected only with the pBI121-GFP suspension as the control group (CK). Transfer the tea seedlings to an artificial climate chamber and keep them in the dark for 12 h. After normal cultivation for 3 days, collect the leaves that were injected with the bacterial suspension, wrap them in aluminum foil, and quickly freeze them in liquid nitrogen. Store them at −80 °C for subsequent analysis.

Gene silencing validation was also conducted. The constructed TRV2-CsHMAs, empty TRV2, and TRV1 plasmids were transformed into the Agrobacterium tumefaciens strain GV3101. Correct positive clones were identified and cultured overnight in YEB liquid medium containing both Kanamycin (Kan) and Rifampicin (Rif) antibiotics. The bacterial cultures were collected and resuspended, and the OD_600_ was adjusted to around 1.5. Next, the treatment group TRV2-CsHMAs resuspension (equal volumes of TRV2-CsHMAs and TRV1 mixed together) and the control group TRV2 resuspension (equal volumes of empty TRV2 and TRV1 mixed together) were incubated in the dark at 28 °C for 3–5 h. Healthy ‘Longjing 43’ tea cuttings were selected. The second and third leaves were gently punctured with the needle of a syringe, and the leaves were infiltrated with the bacterial suspension using a vacuum pump until the suspension spread across the entire leaf surface. After infiltration, the plants were rinsed under running water to remove any surface bacterial suspension and returned to the hydroponic solution for recovery. Subsequently, the tea seedlings were managed under normal cultivation conditions. Once the roots had developed, the tea seedlings that were confirmed to be positive for transient interference by qRT-PCR (both the TRV2-CsHMAs and the empty vector control) were subjected to Cd treatment.

### 4.7. Data Processing and Statistical Analysis

Statistical analyses were performed on the changes in Cd content in tea leaves, stems, and roots, as well as on data pertaining to secondary metabolites, enzyme activities, photosynthetic parameters, and quantitative expression of tea plant genes. These analyses were conducted using Microsoft Excel 2016 and R version 2.2.1. Additionally, Origin 8.5 (OriginLab Corporation, Northampton, MA, USA) was utilized to conduct analysis of variance (ANOVA) on the aforementioned datasets [50].

## 5. Conclusions

Low pH values and high Cd concentrations increase the Cd content in tea, reduce the content of secondary metabolites, inhibit the growth and development of tea, weaken the photosynthetic system, and activate the antioxidant system of tea. *CsHMA2* is involved in regulating the transport of Cd^2+^ in tea. Low pH values enhance the ability of *CsHMA2* to transport Cd^2+^, and overexpression of *CsHMA2* increases the transport capacity of Cd^2+^, while silencing *CsHMA2* reduces the transport capacity of Cd^2+^. The findings of this study not only help to understand how tea adapts and regulates its physiological processes in acidic environments but also provide important theoretical foundations and technical guidance for future soil improvement in tea gardens, heavy metal pollution control, and optimization of tea quality. Meanwhile, it is crucial to develop soil amendments for acidified soils; explore efficient and environmentally friendly application techniques to adjust the pH value of soils; and improve the availability of mineral elements, which is of great significance for the healthy development of soil.

## Data Availability

The original contributions presented in this study are included in the article/Supplementary Materials.

## Supplementary Materials

The following supporting information can be downloaded at: https://www.mdpi.com/article/10.3390/plants14040570/s1, Figure S1. Effects of different pH and Cd treatment on the contents of metabolites in young leaves. Note: (a) AA content; (b) TP content; (c) CAF content; (d) SS content; (e) EGC content; (f) EC content; (g) EGCG content; (h) ECG content; (i) GCG content; (j) GC content. All data were the mean ± standard deviation of 3 biological replicates, each containing 3 technical replicates. Different letters indicated statistically significant differences (*p* < 0.05). Figure S2. Effects of different pH and Cd concentrations on tea plant growth index. Note: (a) Top bud number; (b) Stem diameter; (c) The elongation of the root; (d) Number of root tip. All data were the mean ± standard deviation of 3 biological replicates, each containing 3 technical replicates. Different letters indicated statistically significant differences (*p* < 0.05). Figure S3. Effects of different pH and Cd concentrations on tea plants. Note: The scale in the figure represents 1 cm. Figure S4. Effects of different pH and Cd concentrations on the phenotype of mature leaves and tea plant chlorophyll. Note: (a) The phenotype of mature leaves (b) The content of chlorophyll a; (c) The content of chlorophyll b; (d) The carotenoid content; (e) The total chlorophyll. All data were the mean ± standard deviation of 3 biological replicates, each containing 3 technical replicates. Different letters indicated statistically significant differences (*p* < 0.05).The scale in the figure represents 1 cm. Figure S5. Pseudo color image of Fv /Fm value under different pH and Cd concentrations. Figure S6. Effects of different pH and Cd concentrations on the stoma of tea. Note: The scale in the figure represents 100 μm. Figure S7. Effects of different pH and Cd concentrations on the ratio of pore length to width in tea (n = 15). Note: * means statistically significant difference, * (*p* < 0.05), ** (*p* < 0.01), *** (*p* < 0.001). Figure S8. Effects of different pH and Cd concentrations on Pro and MDA content of tea plants. Note: (a) Pro content; (b) MDA content. Figure S9. Phylogenetic analysis of HMA gene family members in *Camellia sinensis*, *Arabidopsis thaliana*, *Oryza sativa*, *Actinidia and Vitis vinifera.* Figure S10. The expression patterns of *CsHMA* genes in stem of tea plants under different pH and Cd concentrations. Figure S11. The expression patterns of *CsHMA* genes in root of tea plants under different pH and Cd concentrations. Figure S12. Subcellular localization of transiently expressed 35::GFP-CsHMA2. Note: 35S::GFP as the control; mRFP-NLS is a nuclear marker; The scale bars is 20 μm. Figure S13. *CsHMA2* gene was over expressed in high concentration of Cd. Note: (a) CsHMA2 gene expression (b) Cd content in leaves (c) Cd content in stems (d) Cd content in roots (e) tea plant phenotype (f) Live fluorescence of tea plant. * (*p* < 0.05), ** (*p* < 0.01), the scale in the figure represents 1 cm. Figure S14. *CsHMA2* gene was silenced in high concentration of Cd. Note: (a) *CsHMA2* gene expression (b) Cd content in leaves (c) Cd content in stems (d) Cd content in roots (e) tea plant phenotype (f) Live fluorescence of tea plant. Figure S15. CsHMA2 gene was silenced in high concentration of Cd under acidification. Note: (a) CsHMA2 gene expression (b) Cd content in leaves (c) Cd content in stems (d) Cd content in roots (e) tea plant phenotype (f) Live fluorescence of tea plant. Table S1. Primers sequences for quantitative qRT-PCR. Table S2. Primers for gene cloning and vector construction.
