# Supplementary material for: The Physiological Responses of Tea to pH and Cd Conditions and the Effect of the CsHMA2 on Cd Transport
Source: Plants (Basel). 2025 Feb 13;14(4):570. doi: 10.3390/plants14040570 (PMC11859789; doi:10.3390/plants14040570)
Supplement: Supplementary file 1 [file plants-14-00570-s001.zip › plants-3397760-supplementary.pdf]

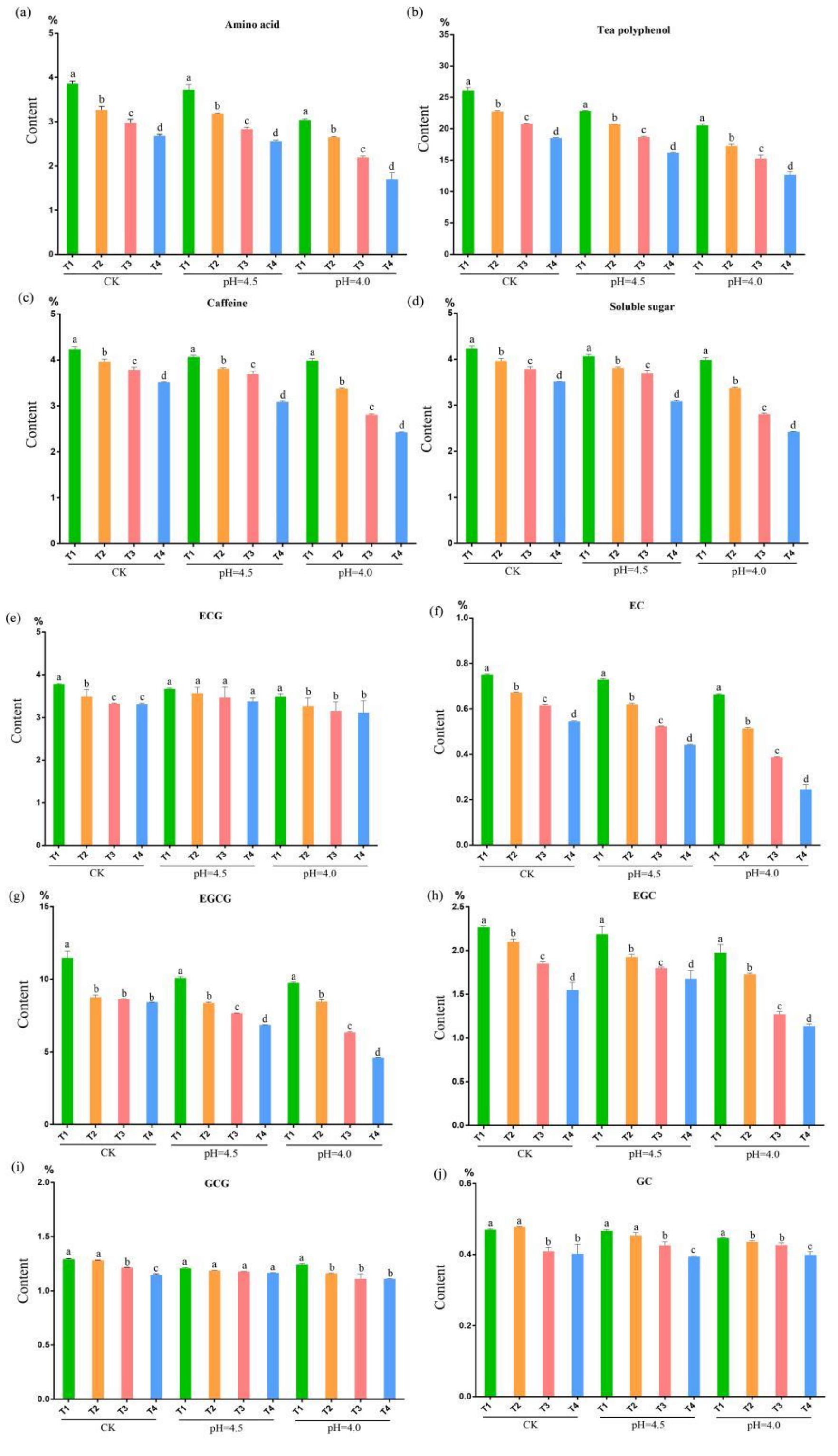

Figure S1. Effects of different pH and Cd treatment on the contents of metabolites in young leaves

Note: (a) AA content; (b) TP content; (c) CAF content; (d) SS content; (e) EGC content; (f) EC content; (g) EGCG content; (h) ECG content; (i) GCG content; (j) GC content. All data were the

mean ± standard deviation of 3 biological replicates, each containing 3 technical replicates. Different letters indicated statistically significant differences ($P < 0.05$).

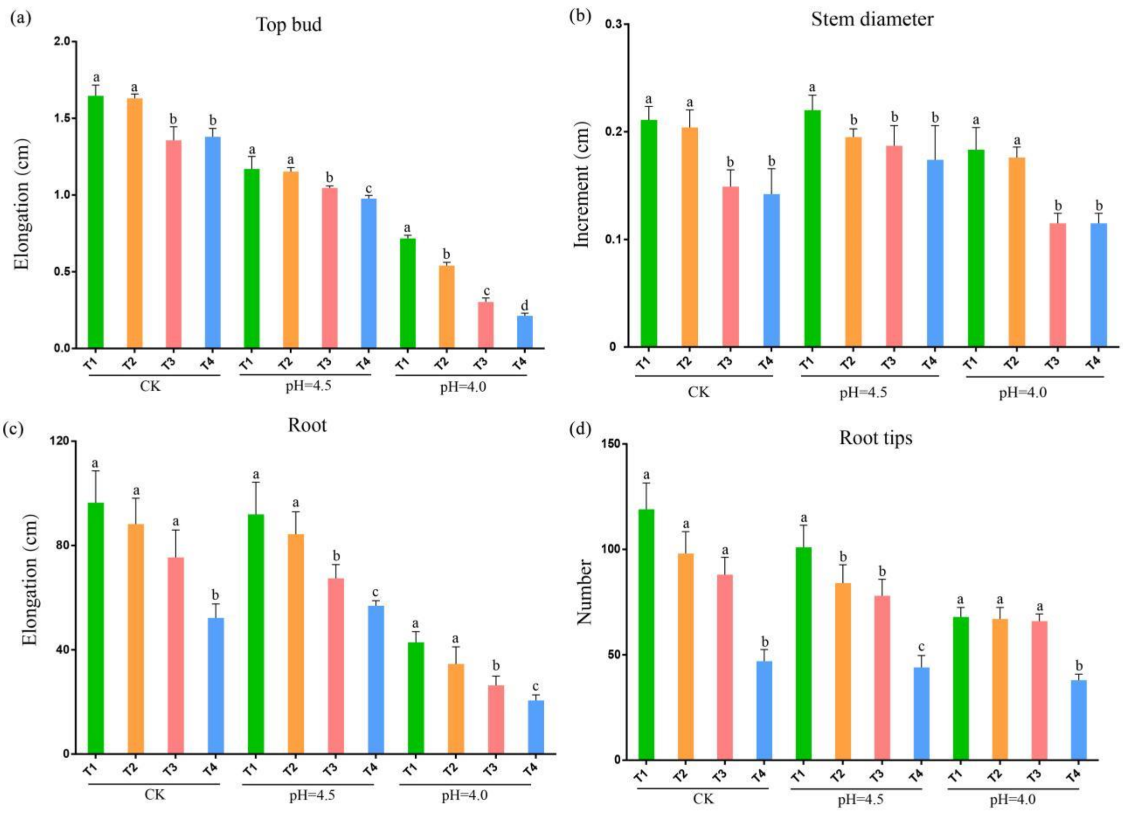

Figure S2. Effects of different pH and Cd concentrations on tea plant growth index

Note: (a) Top bud number; (b) Stem diameter; (c) The elongation of the root; (d) Number of root tip. All data were the mean ± standard deviation of 3 biological replicates, each containing 3 technical replicates. Different letters indicated statistically significant differences ($P < 0.05$).

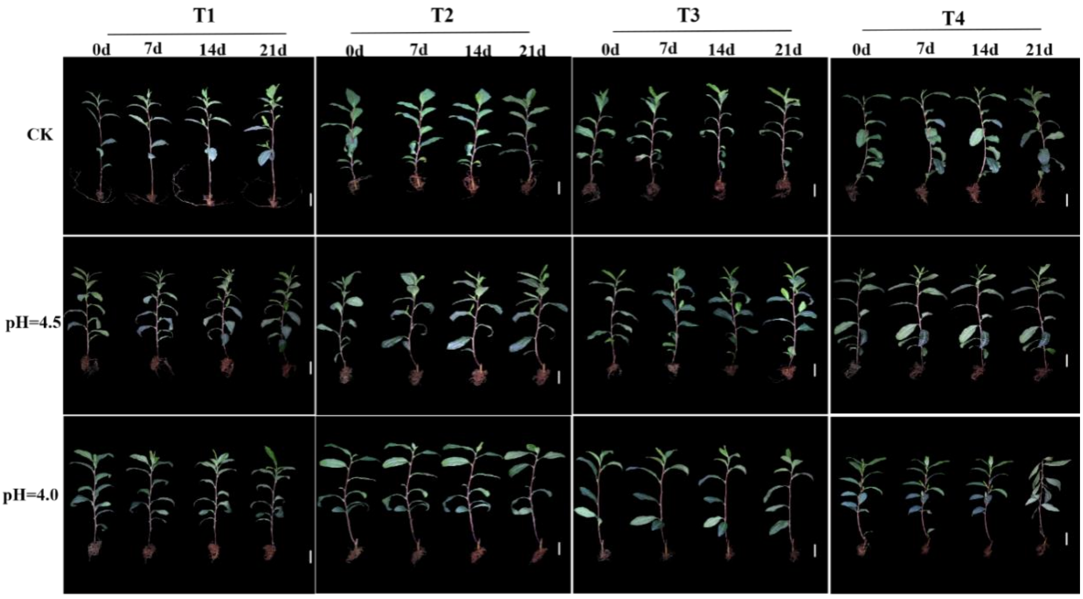

Figure S3. Effects of different pH and Cd concentrations on tea plants

Note: The scale in the figure represents 1 cm.

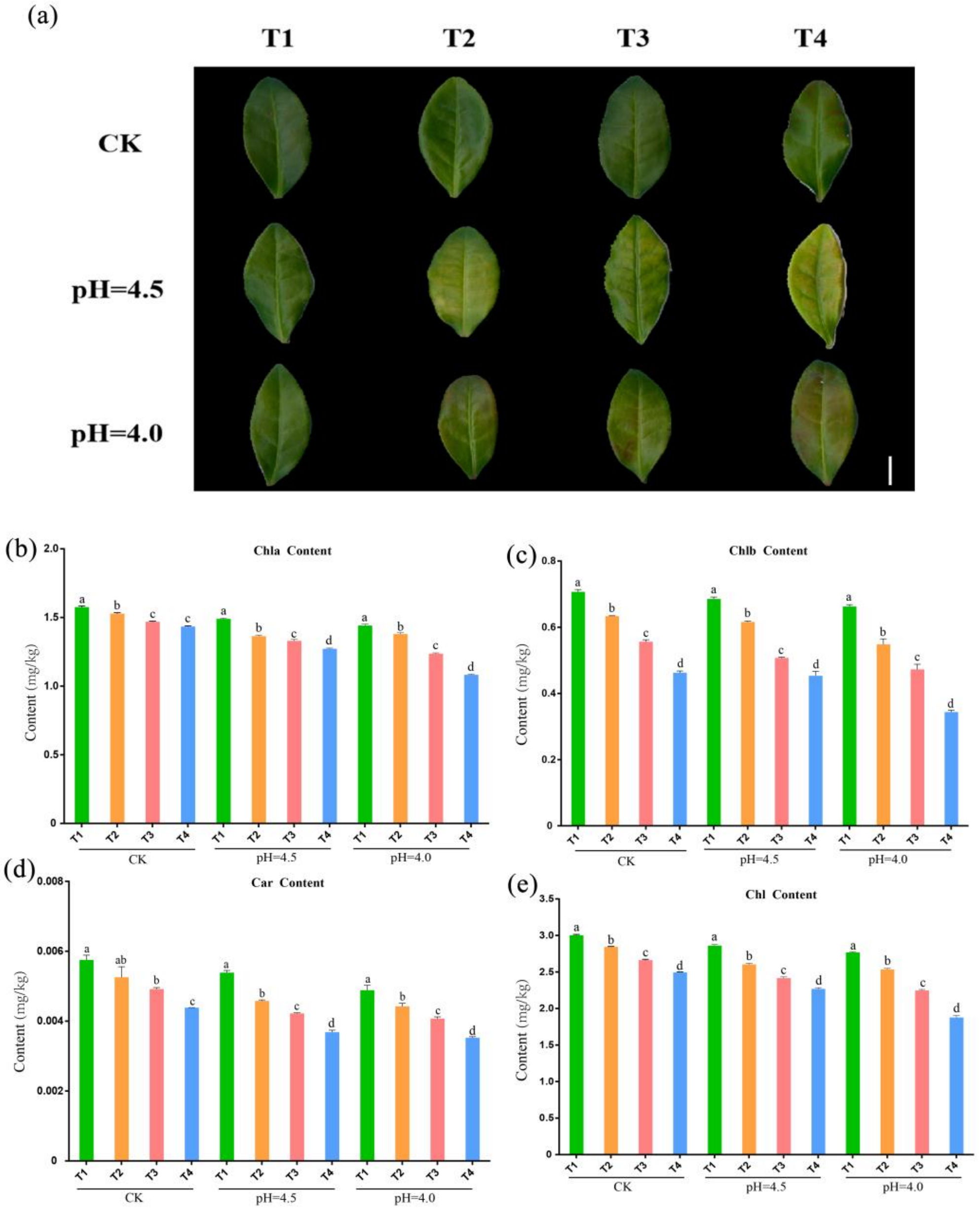

Figure S4. Effects of different pH and Cd concentrations on the phenotype of mature leaves and tea plant chlorophyll

Note: (a) The phenotype of mature leaves (b) The content of chlorophyll a; (c) The content of chlorophyll b; (d) The carotenoid content; (e) The total chlorophyll. All data were the mean ± standard deviation of 3 biological replicates, each containing 3 technical replicates. Different letters indicated statistically significant differences ($P < 0.05$).The scale in the figure represents 1 cm.

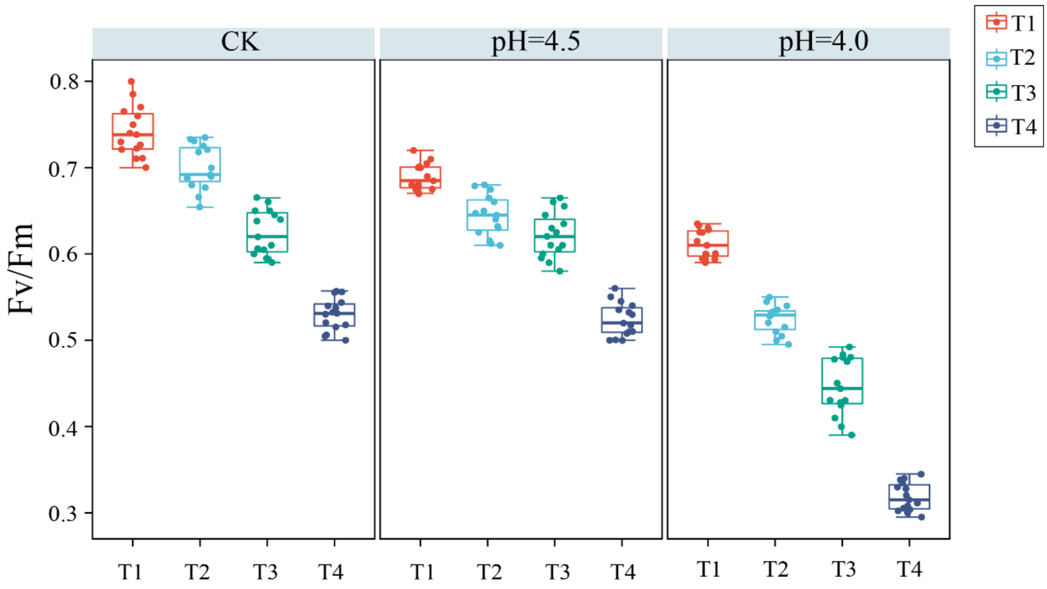

Figure S5. Pseudo color image of Fv /Fm value under different pH and Cd concentrations. (n=15)

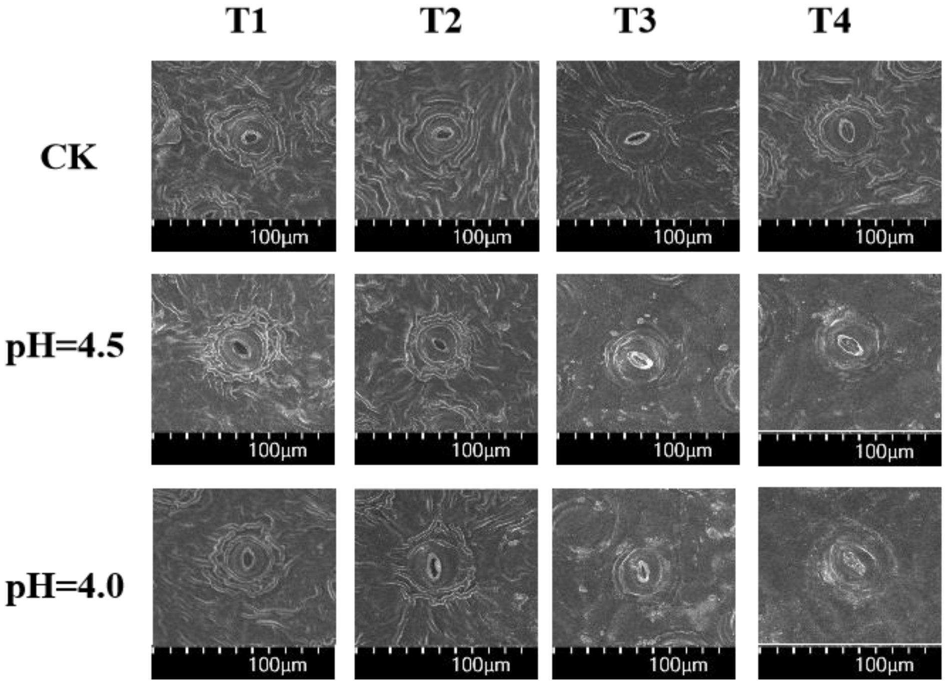

Figure S6. Effects of different pH and Cd concentrations on the stoma of tea

Note: The scale in the figure represents 100 μm.

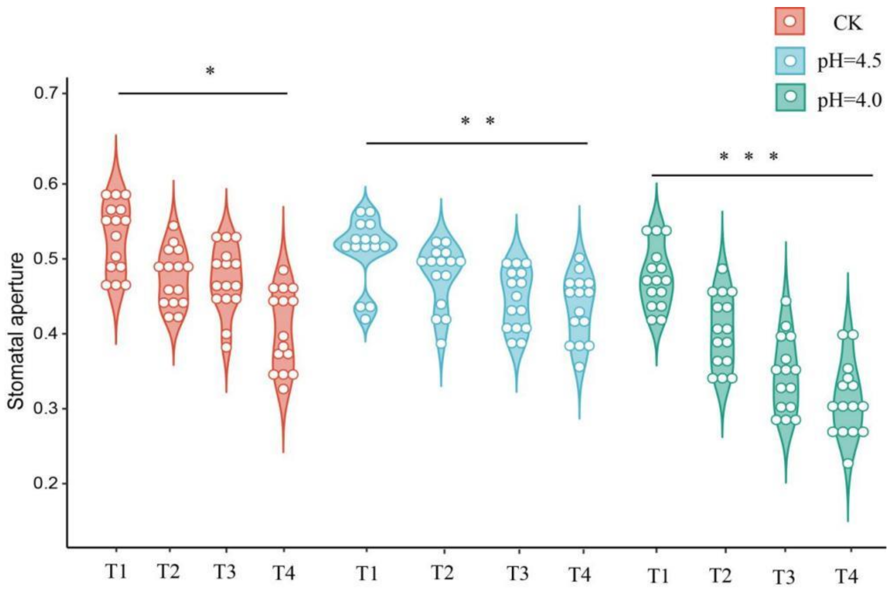

Figure S7. Effects of different pH and Cd concentrations on the ratio of pore length to width in tea (n=15)

Note: * means statistically significant difference, * ($P < 0.05$), ** ($P < 0.01$), *** ($P < 0.001$).

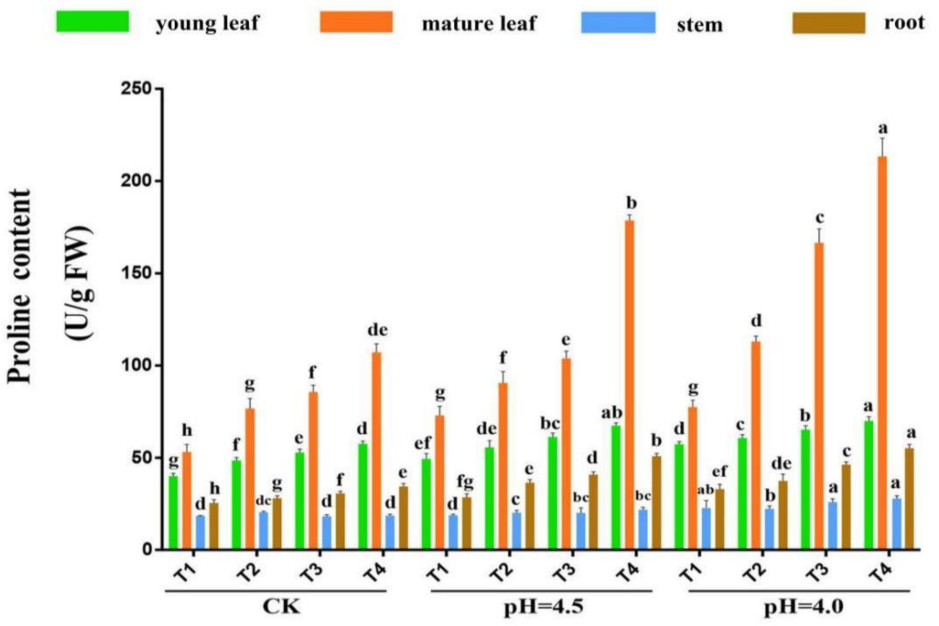

(a)

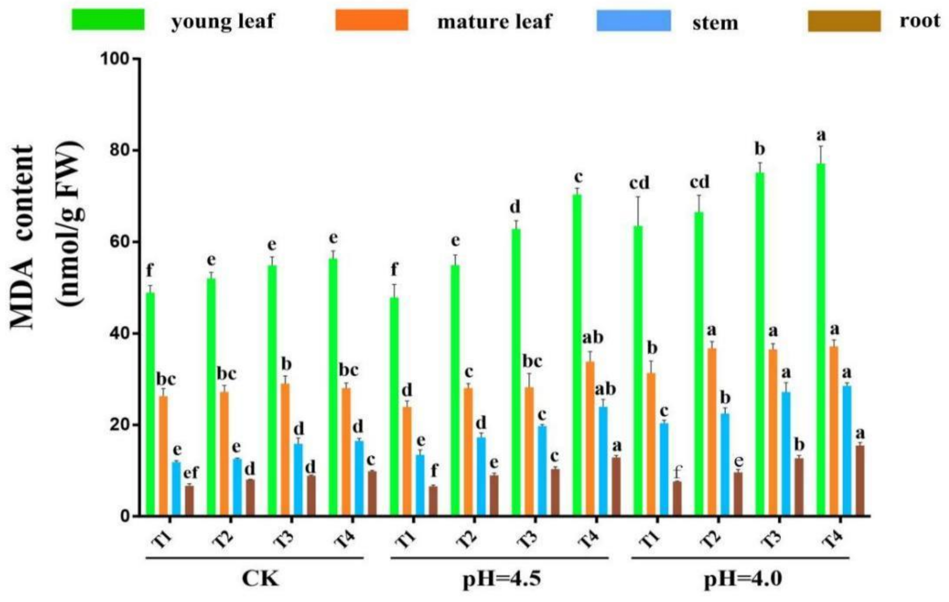

(b)

Figure S8. Effects of different pH and Cd concentrations on Pro and MDA content of tea plants

Note: (a) Pro content; (b) MDA content.

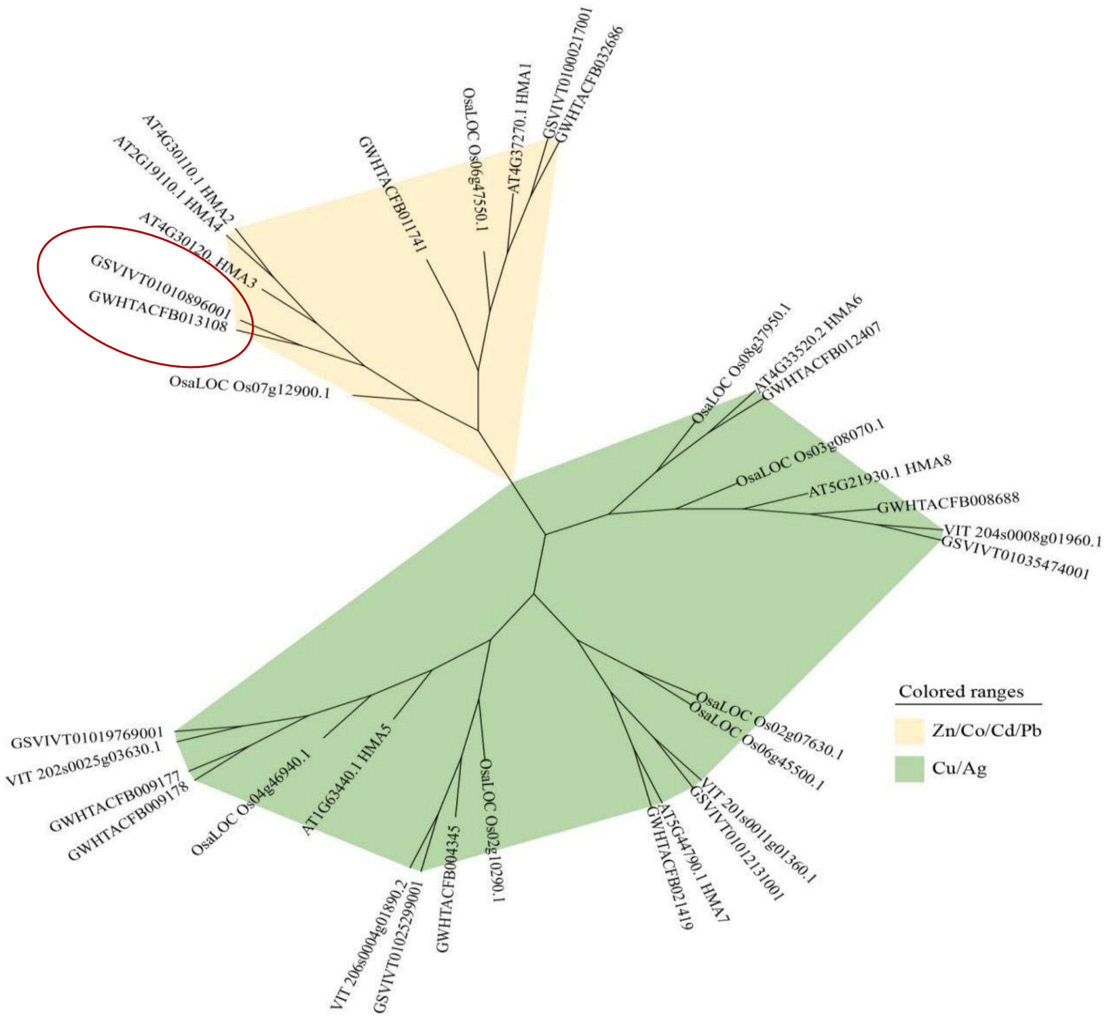

Figure S9. Phylogenetic analysis of HMA gene family members in *Camellia sinensis*, *Arabidopsis thaliana*, *Oryza sativa*, *Actinidia and Vitis vinifera.*

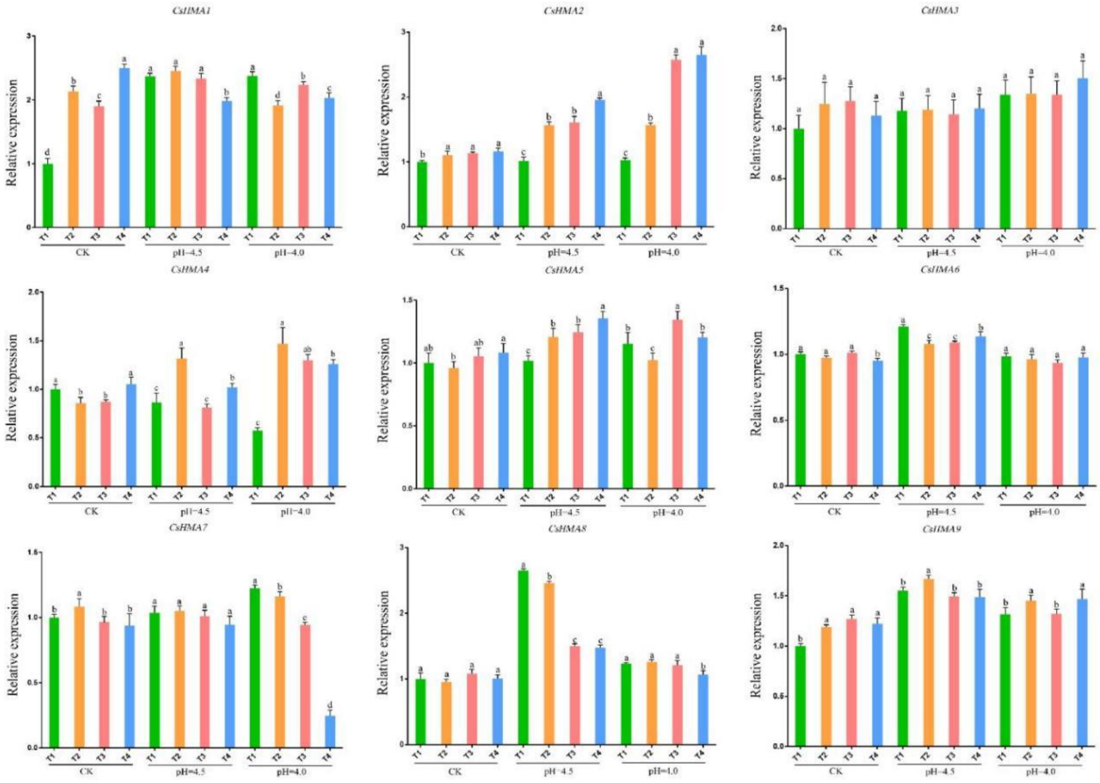

Figure S10. The expression patterns of *CsHMA* genes in stem of tea plants under different pH and Cd concentrations

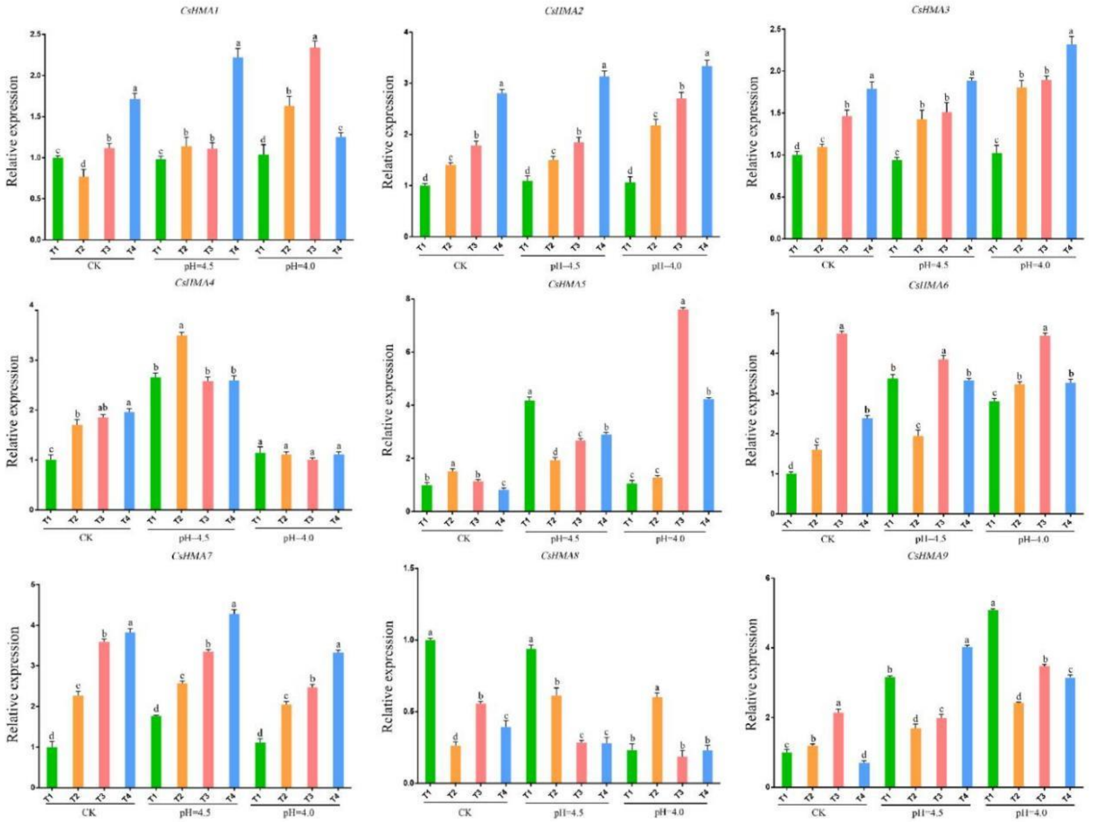

Figure S11. The expression patterns of *CsHMA* genes in root of tea plants under

different pH and Cd concentrations

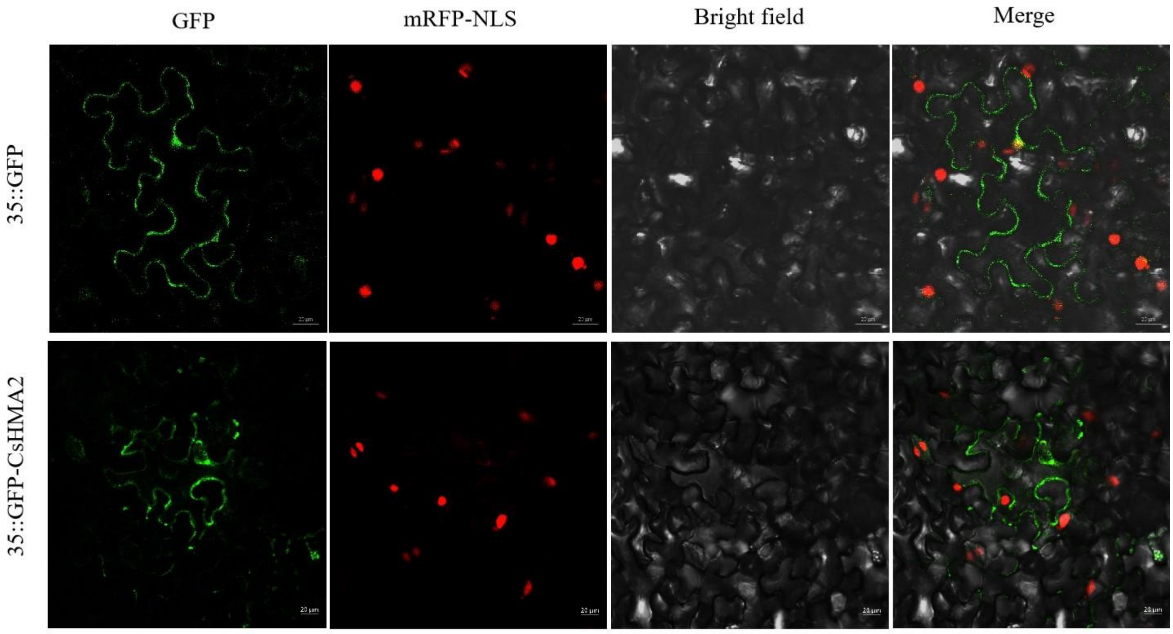

Figure S12. Subcellular localization of transiently expressed 35::GFP-CsHMA2

Note: 35S::GFP as the control; mRFP-NLS is a nuclear marker; The scale bars is 20 μm.

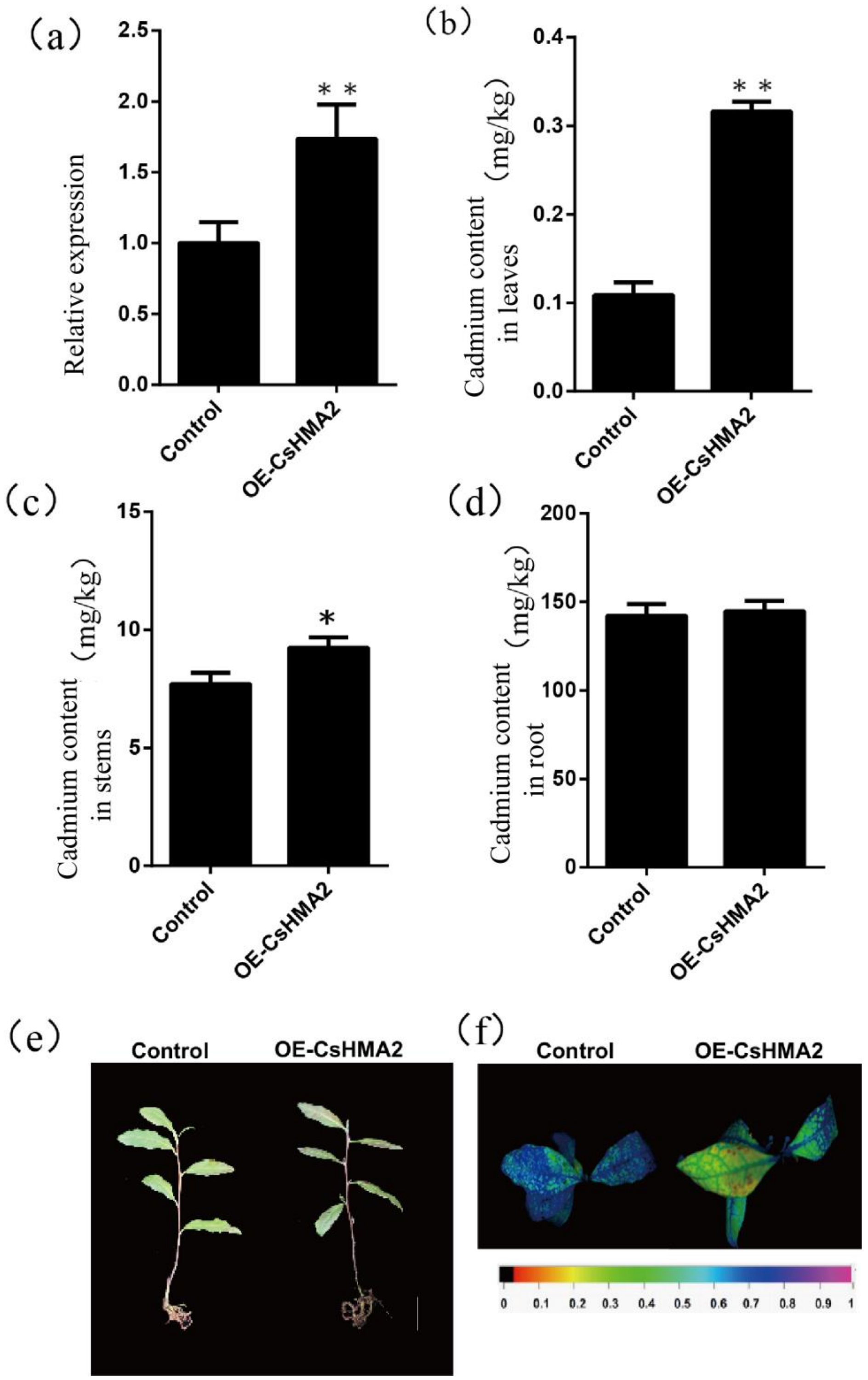

Figure S13. *CsHMA2* gene was over expressed in high concentration of Cd

Note: (a) CsHMA2 gene expression (b) Cd content in leaves (c) Cd content in stems (d) Cd content in roots (e) tea plant phenotype (f) Live fluorescence of tea plant. * (P<0.05), ** (P<0.01), the scale in the figure represents 1 cm.

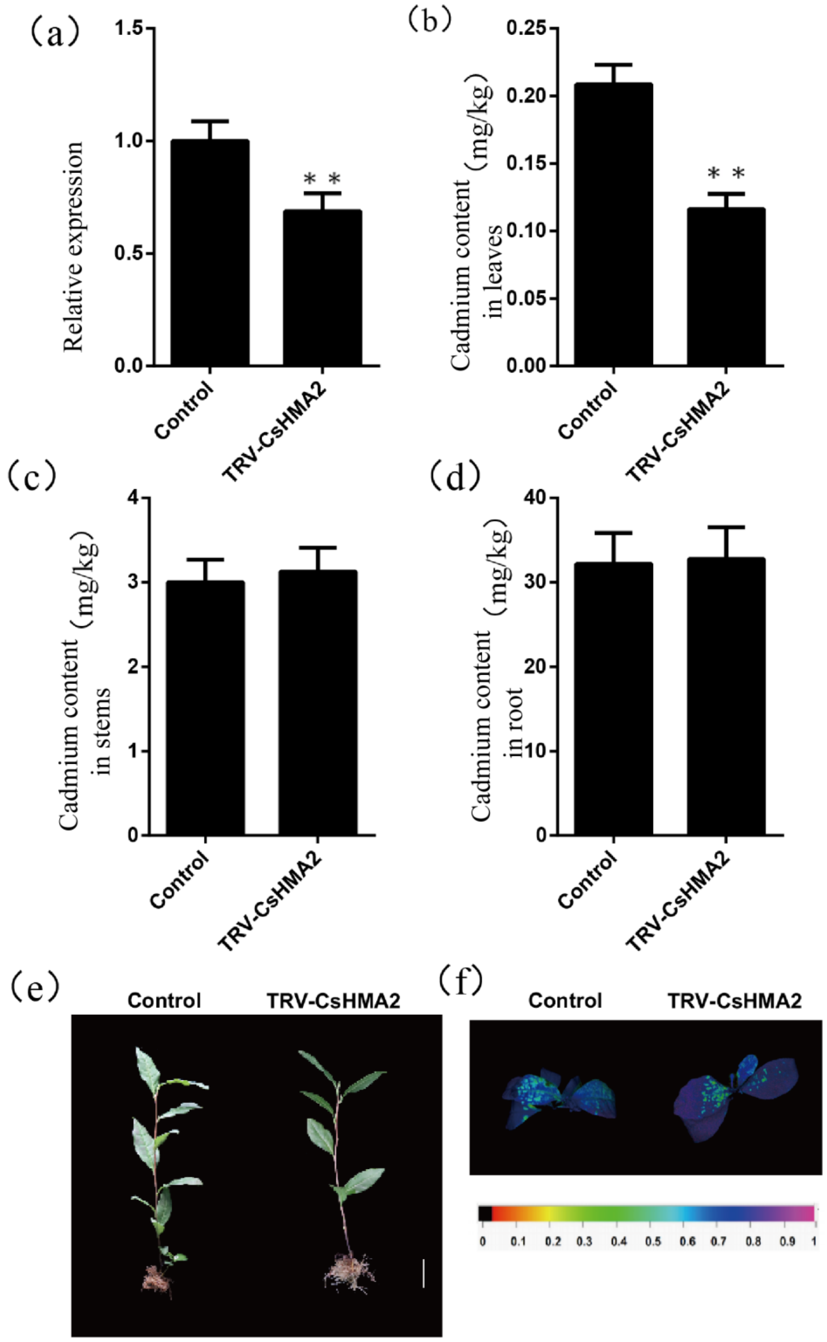

Figure S14. *CsHMA2* gene was silenced in high concentration of Cd

Note: (a) *CsHMA2* gene expression (b) Cd content in leaves (c) Cd content in stems (d) Cd content in roots (e) tea plant phenotype (f) Live fluorescence of tea plant.

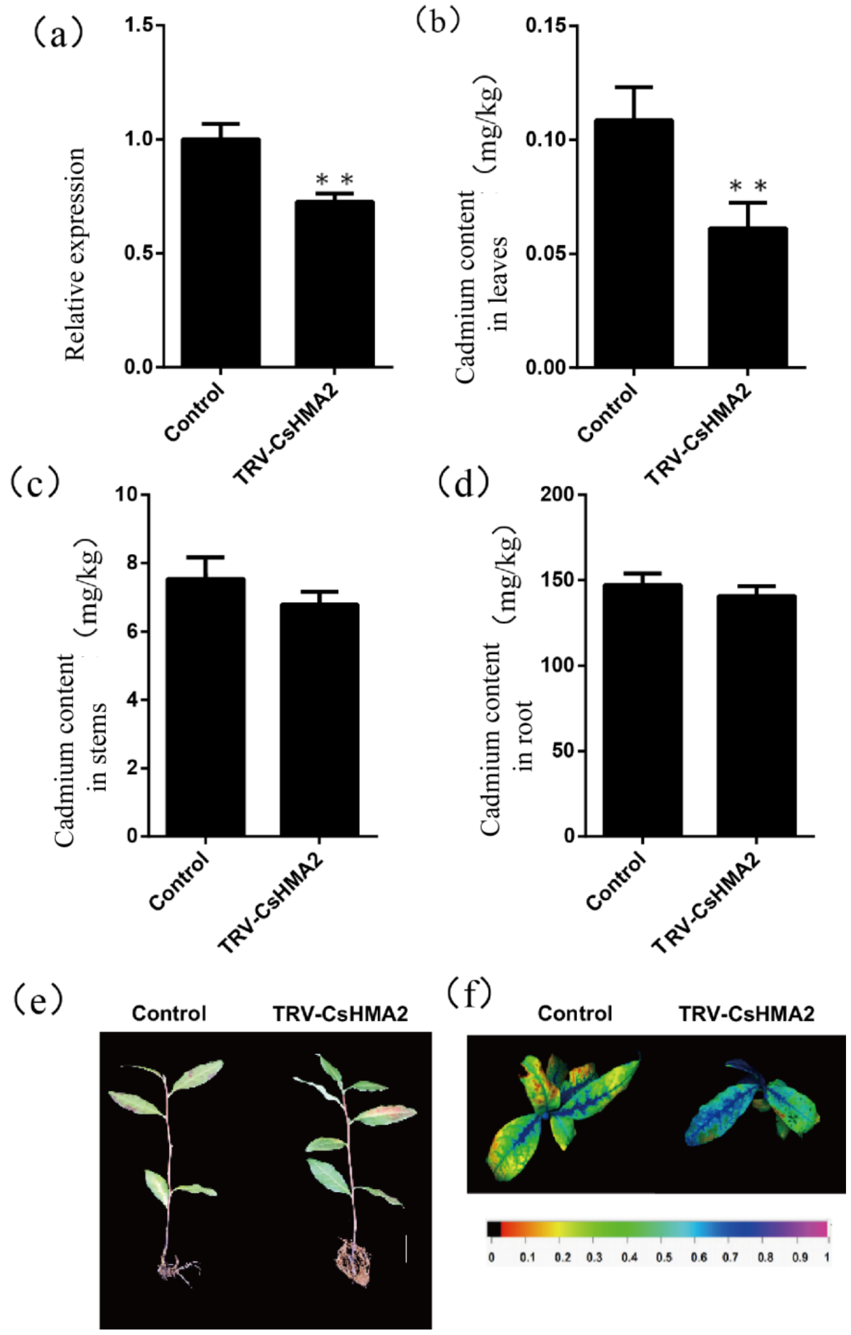

Figure S15. CsHMA2 gene was silenced in high concentration of Cd under acidification

Note: (a) CsHMA2 gene expression (b) Cd content in leaves (c) Cd content in stems (d) Cd content in roots (e) tea plant phenotype (f) Live fluorescence of tea plant.

Table S1. Primers sequences for quantitative qRT-PCR

Gene name	Gene ID	Primers (5’-3’)
CsHMA1-F	GWHTACFB032686	CCTCTCGTTGGGGTAGGAGT
CsHMA1-R	GWHTACFB032686	CAAAGGCTGCAAGAGCCATC
CsHMA2-F	GWHTACFB013108	GATGTTGGGACGTGTTTGGC
CsHMA2-R	GWHTACFB013108	TGGAGGCTTGGACGTTGTTT
CsHMA3-F	GWHTACFB011741	TCTTGGTGAAGAACCCTCGC
CsHMA3-R	GWHTACFB011741	GCCTGTGCTCAACCCGATTA
CsHMA4-F	GWHTACFB009178	GTCAAGTTTCTGTCGCTGGC
CsHMA4-R	GWHTACFB009178	CCTCCGAACCCTCCATGTTC
CsHMA5-F	GWHTACFB009177	TAAGTTTCTGTCGCTGGCGT
CsHMA5-R	GWHTACFB009177	CCTCCGAACCCTCCATGTTC
CsHMA6-F	GWHTACFB012407	CGAGTTTAAGAGTCCCAAGCCA
CsHMA6-R	GWHTACFB012407	CACACTGCAAGCGAGACCTA
CsHMA7-F	GWHTACFB021419	GGCCGATGTGGAAGAAGACA
CsHMA7-R	GWHTACFB021419	TATAGTGAACTGCCCCACCAG
CsHMA8-F	GWHTACFB008688	ACCAGGACGAGGTCTCACT
CsHMA8-R	GWHTACFB008688	AACCAAGAAGCATTACCGGCT
CsHMA9-F	GWHTACFB004345	GCAACCCCTACAGCAGTCAT
CsHMA9-R	GWHTACFB004345	CTTGGAAAAGAGCACGGCAC
β-actin-F		GCCATCTTTGATTGGAATGG
β-actin-R		GGTGCCACAACCTTGATCTT

Table S2. Primers for gene cloning and vector construction

Gene name	Primers (5’-3’)
CsHMA2-F	TGGCTTGTGGTGTATTGCTTTTGTTATCC
CsHMA2-R	CTCAATGATAATCTTTGTTAATTCTCCTCCG
CsHMA2-TRV-F	AAGGTTACCGAATTCTCTAGATGGCTTGTGGTGTATTGCTTTTGTTATCC
CsHMA2-TRV-R	CGTGAGCTCGGTACCGGATCCCTCAATGATAATCTTTGTTAATTCTCCTCCG
CsHMA2-PBI-F	GAGAACACGGGGGACTCTAGATGGCTTGTGGTGTATTGCTTTTGTTATCC
CsHMA2-PBI-R	GGACTGACCACCCGGGGATCCCTCAATGATAATCTTTGTTAATTCTCCTCCG
